# Manganese Promoted (Bi)carbonate Hydrogenation and Formate Dehydrogenation: Toward a Circular Carbon and Hydrogen Economy

**DOI:** 10.1021/acscentsci.2c00723

**Published:** 2022-10-19

**Authors:** Duo Wei, Xinzhe Shi, Peter Sponholz, Henrik Junge, Matthias Beller

**Affiliations:** † Leibniz-Institut für Katalyse e.V., Albert-Einstein-Str. 29a, 18059Rostock, Germany; ‡ APEX Energy Teterow GmbH, Hans-Adam-Allee 1, 18299Rostock-Laage, Germany

## Abstract

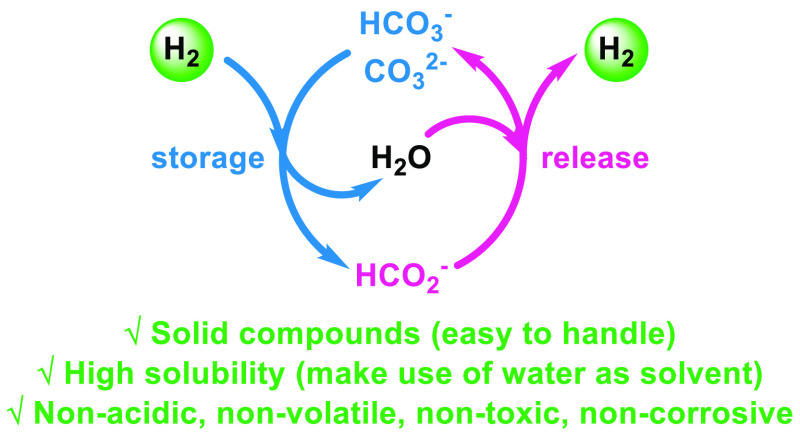

We report here a feasible hydrogen storage and release process by interconversion of readily available (bi)carbonate and formate salts in the presence of naturally occurring α-amino acids. These transformations are of interest for the concept of a circular carbon economy. The use of inorganic carbonate salts for hydrogen storage and release is also described for the first time. Hydrogenation of these substrates proceeds with high formate yields in the presence of specific manganese pincer catalysts and glutamic acid. Based on this, cyclic hydrogen storage and release processes with carbonate salts succeed with good H_2_ yields.

## Abstract 2

Using readily available raw materials (bi)carbonate and formate salts, we report reversible chemical H_2_ storage and release catalyzed by Mn in the presence of naturally occurring α-amino acids.

## Introduction

In contrast to the traditional fossil-based linear economy, the circular economy is a model of production and consumption which involves reducing, reusing, and recycling, aiming to tackle the global resource shortage. Following such a concept, human economy activity and life quality can be sustained and improved while minimizing consumption of fossil resources and emission of wastes.^1^ Efficient and economic carbon valorization is a substantial practice for the circular carbon economy.^2^ Most research efforts, today and in the past, have focused on the fixation of gaseous CO_2_, where a high concentration and pressure of CO_2_ is commonly required, for instance, in carbon capture and utilization (CCU), and direct air capture (DAC) processes,^3−5^ making it expensive to catch carbon back into the ground at a meaningful scale.^6^ Alternatively, improved carbon utilization might be provided with the largely available solid bicarbonate and carbonate salts, which are easily produced from CO_2_/base or so-called “carbonate factories” in nature.^7^ The latter systems are widely used in the scenarios of construction, health and diet, agriculture and aquaculture, household cleaning, and pollution mitigation.^8,9^ Due to their inherent advantages, carbonate and to a lesser extent bicarbonate salts are expected to play considerable roles in the present and future circular carbon economy.

Apart from the production of urea,^10^ cyclic and polycarbonates,^11−13^ etc., which possess the same oxidation state as (bi)carbonates, their effective reduction is a key step in not only sustainable production of chemicals and fuels but also further implementation of chemical hydrogen storage and release via reversible hydrogenation of bicarbonates.^14−16^ As clean energy carrier, H_2_ is attracting increasing attention, owing to its sustainable production from renewable resources and green combustion in fuel cells providing only water and energy.^17−20^ To avoid transportation and handling of gaseous H_2_ with low volumetric storage density, solid or liquid organic H_2_ carriers have emerged as substitutes to realize the on-demand reversible chemical H_2_ storage and release.^21−26^
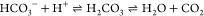
1
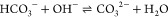
2
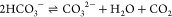
3
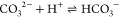
4

**Figure 1:**
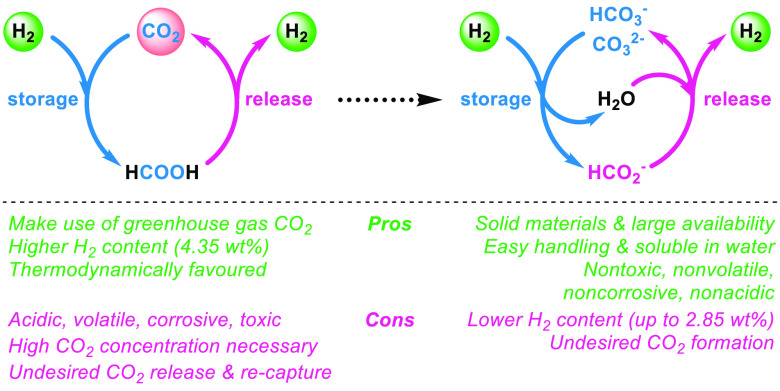
Different concepts of chemical hydrogen storage and release based on the interconversion of CO_2_/formic acid (left) and (bi)carbonate/formate (right).

Besides the well-known CO_2_/formic acid (FA) based hydrogen storage system (Figure 1, left),^27−35^ the equivalent bicarbonate/formate cycle has been investigated (Figure 1, right).^15,36−41^ Basically, all of these works make use of less available precious metal catalysts for the corresponding (de)hydrogenation reactions. One of the challenges applying bicarbonates in chemical H_2_ storage-release cycles is the undesired formation of carbonates and CO_2_ under specific conditions [eqs 1–3], which limits the theoretical capacity of such a hydrogen storage-release system after the initial cycle.^42^

The hydrogen contents in FA (4.35 wt %) and formate salts (1.02–2.85 wt %) are comparable to H_2_ storage alloys, e.g., magnesium hydrides (1–6 wt %).^43^ Nonetheless, the inferior hydrogenation/dehydrogenation kinetics, life cycle, and harsh operation conditions (300–500 °C) of such alloys make them currently not appropriate for most applications.^43,44^

Due to the lower reactivity of the carbonyl group in carbonates compared to CO_2_ or bicarbonates, their catalytic hydrogenation is more demanding. Consequently, the transformation of inorganic carbonate to formate salts is rarely reported. Examples generally proceed in low yields (<10%) and poor selectivity using glycerol,^45^ hydrosilanes,^46^ and H_2_^47,48^ as reducing agents. In addition, both our group^36^ and Joó et al.^49^ reported the Ru catalyzed hydrogenation of carbonate salts in the presence of CO_2_. Furthermore, a transition-metal-free method was reported for the carbonate hydrogenation to a mixture of formate, acetate, and oxalate with H_2_/CO_2_ (30/30 bar) at 230–320 °C.^50^

The present situation and our general interest in hydrogen storage technologies prompted us to investigate the interconversion of (bi)carbonate and formate salts as a general H_2_ storage-release method.

## Results and Discussion

### Hydrogenation of Bicarbonate to Formate

**Figure 2:**
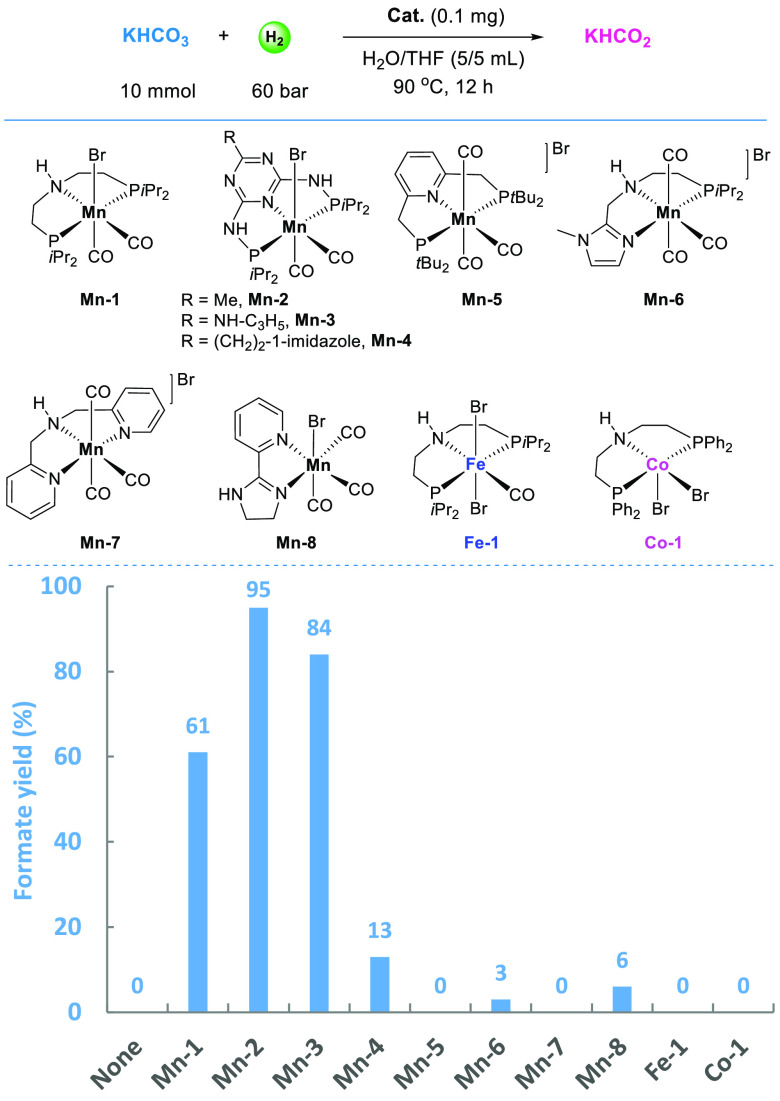
Hydrogenation of potassium bicarbonate to potassium formate catalyzed by base metal complexes. Standard conditions: KHCO_3_ (10 mmol), catalyst (0.1 mg), H_2_O/THF (5/5 mL), H_2_ (60 bar), 90 °C, 12 h. Yield of formate is calculated by (mmol formate)/(mmol KHCO_3_) × 100%.

We started the bicarbonate-to-formate transformation in water and THF (v:v = 1:1) as co-solvent, H_2_ (60 bar), 90 °C and 12 h (Figure 2). Apparently, in the absence of any catalyst, no formate was formed. Among all the tested complexes, the most successful one was identified as **Mn-2** bearing a methyl group at the triazine-based pincer ligand, leading to formate in 95% yield (TON 55,000, Figures S1–S2).

At this point, it is worthwhile mentioning that mainly noble metals, Fe,^48,51−58^ Co,^59^ and Ni,^60,61^ catalysts have been reported in bicarbonate hydrogenation. Manganese complexes have been rarely used in this area, with limited examples in CO_2_ hydrogenation^62−67^ and FA dehydrogenation,^68−71^ despite its nature of abundance, nontoxicity, biocompatibility, and environmental friendliness.^72−77^ In addition to **Mn-2**, **Mn-1** and **Mn-3** have produced formate in 61% and 84% yields, respectively while other Mn-pincer/bidentate complexes and Fe and Co analogues gave formate in yields up to 13%. Notably, no additional metal base promoter, e.g., potassium *tert*-butoxide, is necessary in the current reaction. Further hydrogenation of the product KHCO_2_ to methanol is not observed under current conditions. Analysis of the gas phase after hydrogenation reactions revealed no detectable CO_2_, CO, or CH_4_, indicating the distinct selectivity of bicarbonate-to-formate transformation catalyzed by the selected manganese complexes. Replacing THF by other organic solvents, e.g., dioxane, triglyme, ethanol, 2-methyl-THF, or only water as a single solvent, resulted in decreased formate yields (2–77%, Figure S3). Trials with three other bicarbonate salts based on Na^+^, Cs^+^, and NH_4_^+^ cations led to moderate formate yields (46–69%) compared to KHCO_3_ (Figure S4).

### Carbonate Hydrogenation to Formate

**Figure 3:**
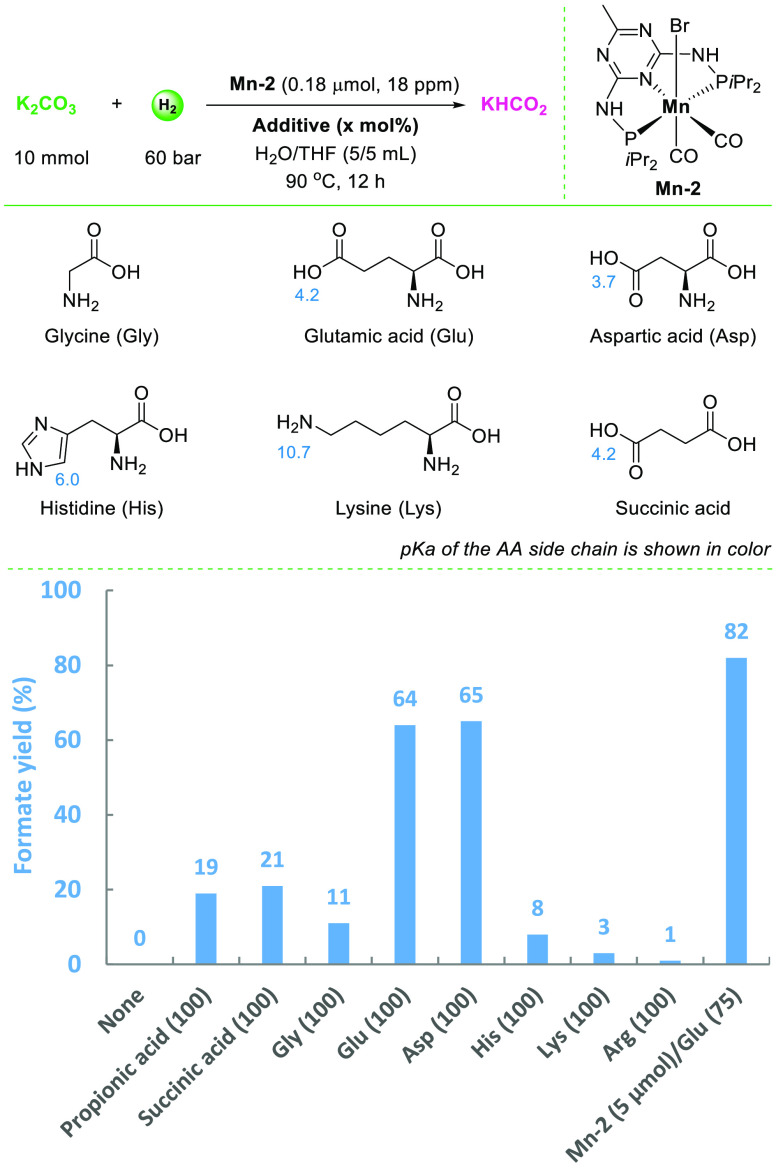
Catalytic hydrogenation of potassium carbonate to potassium formate. Conditions: K_2_CO_3_ (10 mmol), **Mn-2** (0.18 μmol), H_2_O/THF (5/5 mL), H_2_ (60 bar), additive, 90 °C, 12 h. Yield of formate is calculated by (mmol formate)/(mmol K_2_CO_3_) × 100%.

After succeeding in the hydrogenation of bicarbonates, we addressed the more desirable transformation of carbonates to formates. Indeed, no hydrogenation of potassium carbonate occurred applying **Mn-2** complexes under the reaction conditions shown in Figure 2, and no formate was detected (Figure 3). To promote this less favored reaction, addition of a carboxylic acid seems logical according to eq 4. Compared to inorganic acids, e.g., HCl, the higher boiling points of carboxylic acids are beneficial to maintain themselves in reaction cycles. Thus, by simply adding propionic acid, some conversion was observed, albeit the formate yield was low (19%). Similarly, testing other dicarboxylic acids as well as α-amino acids (AAs) revealed some reactivity. Surprisingly, the structure of the AA has a strong influence on the formate yield. While in the presence of the simplest AA glycine (Gly) a 11% formate yield was observed, AAs bearing acidic side chains, e.g., glutamic acid (Glu) and aspartic acid (Asp), led to much higher formate yields (up to 65%). In contrast, when utilizing basic AAs histidine (His), lysine (Lys), and arginine (Arg), the yields of formate dropped drastically. Apparently, a proper acidic media is important for higher formate efficiency.

Further investigations focused on Glu as the best promoter. Variation of the Glu loading (0–150 mol %) led to different formate yields (up to 65%, Figure S6). Besides, CO_2_ was detected in the gas mixture after hydrogenation reactions which derived from carbonate salt. The optimal loading of Glu was found at 75 mol % based on the formate yield. The Li^+^, K^+^, Rb^+^, and NH_4_^+^ based carbonate salts gave the best formate yields among the tested nine different carbonate species (61–78%, Figure S7). Finally, applying 5 μmol of **Mn-2** catalyst, formate was produced in 82% yield starting from K_2_CO_3_ (Figure S8). Alternatively, using CO_2_ (10 bar) instead of acid additives, a comparable formate yield (83%) was obtained (Figure S19).

The positive influence of Glu on the formate yield is explained by its dual function: (a) sufficient acidity and (b) carbon dioxide capture ability. Indeed, control reactions between K_2_CO_3_ and different acids (Table S1) showed that addition of propionic acid led to bicarbonate as the main product (71%) with 8% CO_2_, while this ratio was reversed in the presence of the stronger succinic acid with 3% bicarbonate and 95% CO_2_. Interestingly, when Glu was mixed with K_2_CO_3_, carbamate species were observed in 26% yield along with bicarbonate (51%) and CO_2_ (16%), indicating the significant CO_2_ capture effect of Glu. Apparently, the formation of carbamate species is substantial to achieve a high yield in the carbonate-to-formate transformation.

### Hydrogen Production from Formate

**Figure 4:**
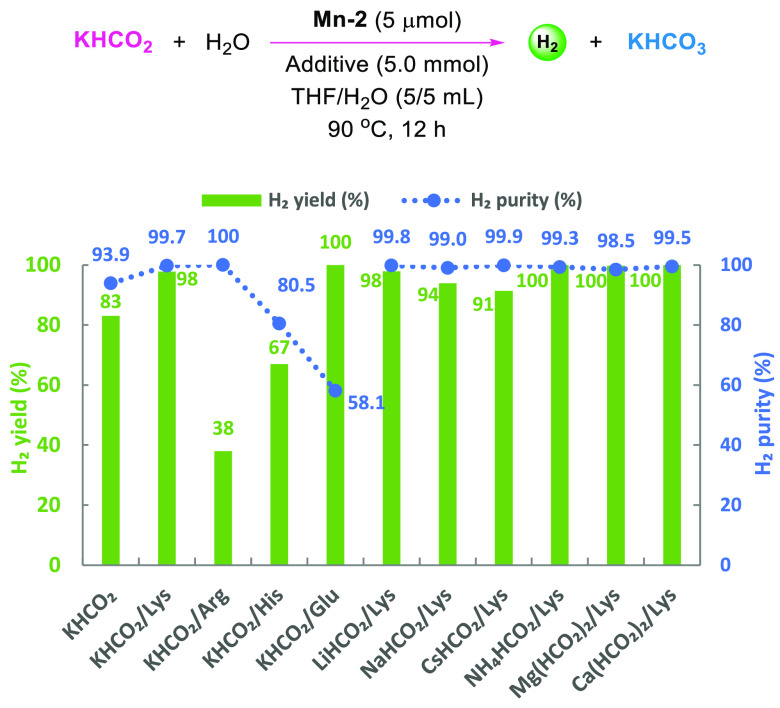
Catalytic hydrogen production from formates. Conditions: formate (5.0 mmol), **Mn-2** (5 μmol), additive (5.0 mmol), H_2_O/THF (5/5 mL), 90 °C, 12 h. Yield of H_2_ is calculated by (mmol H_2_)/(mmol formate) × 100%. The dotted lines serve as guides to the eye.

For the development of a round-trip hydrogen storage system, the release of hydrogen is important, too. Hence, after having suitable conditions for hydrogenate (bi)carbonates, we investigated hydrogen production from formate under similar reaction conditions. In the absence of any additive, **Mn-2** among all the tested catalysts gave the best H_2_ yield (74%) and H_2_ purity (94.5%) besides CO_2_, which results from the decomposition of bicarbonate (Figure S9). To promote both the H_2_ yield and purity, the effect of amino acid additives was investigated (Figure 4). After an equimolar amount of Lys^67,78^ was introduced to KHCO_2_, a quantitative H_2_ yield was found with more that 99% purity. Replacing Lys by Arg or His, the yield of H_2_ dropped significantly. Interestingly, a quantitative yield of H_2_ was obtained, albeit with a much higher CO_2_ ratio (41.9%) by using Glu due to its increased acidity.

In the absence of Lys, hydrogen was produced generally in low purity (67–95%) applying various formate salts based on Li^+^, Na^+^, K^+^, Cs^+^, NH_4_^+^, Mg^2+^, and Ca^2+^ cations (Figure S10). In contrast, promising results with both high H_2_ yield (>91%) and purity (>98%) were obtained in the presence of Lys and the above-mentioned formate salts (Figure 4). According to eq 3, carbonates are supposed to capture the released CO_2_ back to bicarbonates. To compare the CO_2_ capture ability between K_2_CO_3_ and Lys, control experiments were performed (Table S4). Due to the presence of amino group in Lys, a significant amount of carbamate species was obtained demonstrating the superior CO_2_ (2 bar) capture ability of Lys (ca. 1.6-fold) compared to K_2_CO_3_ within 0.5 h.

### Reversible H_2_ Storage and Release Based on Bicarbonate/Formate Pair

**Figure 5:**
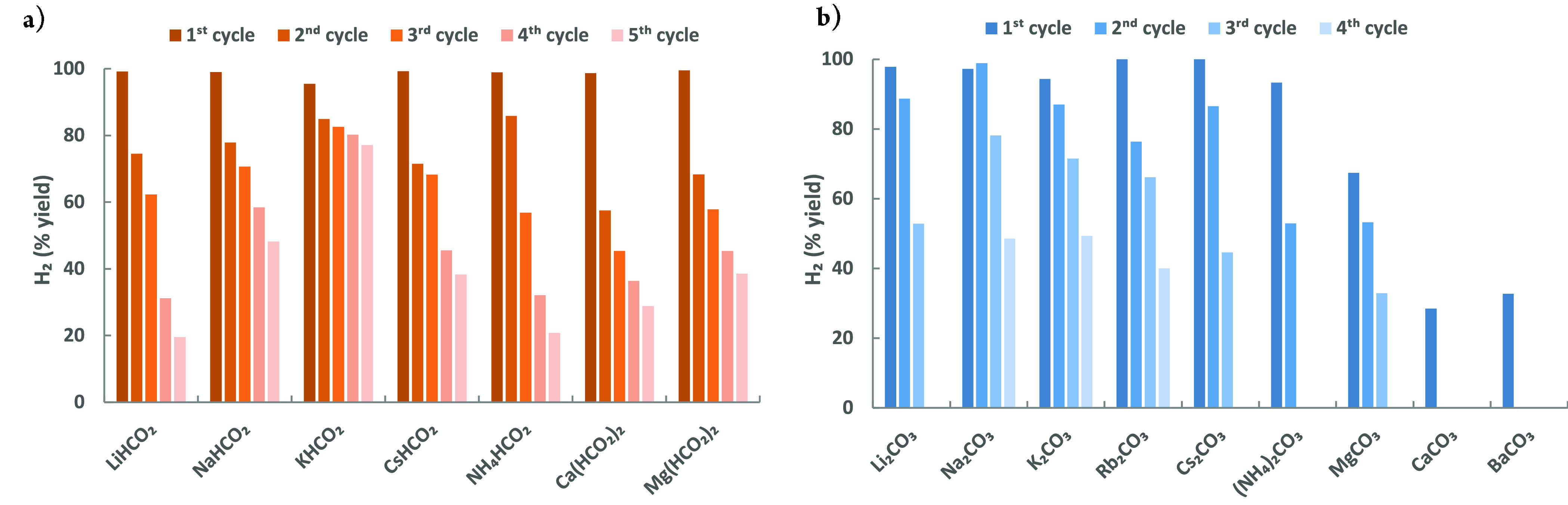
H_2_ storage-release cycles via interconversion of (a) bicarbonate/formate and (b) carbonate/formate. Standard conditions: (a) formate/Lys (5.0/5.0 mmol); (b) carbonate/Glu (5.0/5.0 mmol), **Mn-2** (5 μmol), H_2_O/THF (5/5 mL), 90 °C, 12 h. H_2_ (60 bar) was applied in the hydrogenation step. H_2_ storage-release cycles start with (a) dehydrogenation and (b) hydrogenation. H_2_ yields of each cycle are calculated based on the initial loading of formate or carbonate salts, respectively (each 5 mmol).

For the implementation of a viable hydrogen storage system, it is necessary to combine the two individual processes, i.e., formate dehydrogenation and bicarbonate hydrogenation and demonstrate the possibility of stable hydrogen storage-release cycles (as illustrated in Figure 1, right). Thus, starting from the selected formate salt, H_2_ was generated in a 100 mL autoclave at 90 °C. After completion of the reaction, a buret was used to collect H_2_, and the autoclave was subjected to hydrogen storage under 60 bar of H_2_ and 90 °C. The over pressure of H_2_ was then released after cooling the reaction mixture to r.t., and the dehydrogenation was repeated in the next cycle. Following this protocol, several reaction systems were compared (Figure 5a, Table S2). Starting from different formate salts based on Li^+^, Na^+^, NH_4_^+^, Mg^2+^, Ca^2+^, and Lys, although quantitative H_2_ yields (>95%) were achieved in the initial dehydrogenation reaction, stepwise decreased yields were observed after several cycles, which is ascribed to the low efficiency in hydrogenation of corresponding bicarbonate salts (Figure S4). Interestingly, compared to other formate salts, applying KHCO_2_, 80% of the initial H_2_ productivity remained after five cycles with >99% H_2_ purity. The formation of the bimetallic Mn–K intermediate^79−82^ is speculated to achieve such a good performance.

### H_2_ Storage and Release Cycles Starting from Carbonate Salts

Based on the relevant results of hydrogenation of carbonate salts, we tested the cyclic performance of H_2_ storage-release starting from K_2_CO_3_ and Glu. The favorable loading of Glu fell on 100 mol % (based on carbonate salts) owing to the high H_2_ yields (up to 94%) and reusability of the catalytic system (Table S3). Indeed, from a K_2_CO_3_ and Glu mixture at a pH of 7.9 (Table S5), not only is less CO_2_ release expected, but also the efficient CO_2_ capture by the amino groups of Glu takes place under such basic conditions. As a result, the amount of bicarbonate and carbamate after the first H_2_ storage and release cycle was measured to be 4.5 mmol (90% yield based on initial loading of K_2_CO_3_, Figure S20). Afterward, additional inorganic carbonates were evaluated under the standard conditions (Figure 5b). Similar to K_2_CO_3_, a good reactivity was also obtained using Na_2_CO_3_ and Rb_2_CO_3_ in four consecutive cycles with up to 100% H_2_ yield. Carbonate salts based on Li and Cs could be reused in at least three cycles. Moreover, it was surprising that the easily available raw material MgCO_3_ could be utilized in the current H_2_ storage systems achieving feasible efficiency (67% H_2_ yield) in the first storage cycle, although decreased yields in the subsequent runs were observed. On the other hand, calcium- and barium-based carbonate salts gave H_2_ yields in up to 33% in the initial cycles, due to their poor solubilities in water (ca. 0.02 mg mL^–1^ at 25 °C).

According to previous reports, FA/formates dehydrogenation^83,84^ and its reverse reactions^62,65,84−86^ could be promoted by acids. In a detailed study of FA dehydrogenation,^83^ the rate limiting step, i.e., decarboxylation of the metal-formate intermediate (M–OOCH) is assisted by Lewis acid (LiBF_4_) or Brønsted acid [Et_3_NH]^+^. This lowers the activation energy of the decarboxylation process, thus improving the reaction rates. In our earlier work,^67,78^ control experiments showed that the presence of an α-amino acid group and an appropriate basic side chain in the amine molecule are both crucial to facilitate the CO_2_ hydrogenation. Therefore, we propose that α-amino acids could promote the H_2_ yield via stabilizing the Mn–OOCH intermediate and accelerating the corresponding decarboxylation process.

## Conclusion

To conclude, we provide a viable Mn promoted reversible hydrogen storage and release method via the interconversion of largely available (bi)carbonate and formate salts under comparably mild reaction conditions. For the first time, low-cost carbonate salts could be applied as part of a H_2_ storage-release system with the help of naturally occurring AA glutamic acid (Glu) as an additive, where the released CO_2_ could be ideally captured by the amino group of Glu and hydrogenated back to formate to close the cycle. Notably, the overall system can operate below 100 °C, making the utilization of so-called “waste heat” possible.^87^ The dehydrogenation step of the resulting formate proceeds smoothly without carbon dioxide liberation in the presence of lysine. This enables hydrogen storage-release applications as shown by several charge–discharge cycles with >80% H_2_ evolution yield and >99% purity applying potassium formate, without reloading of catalyst, solvent, and hydrogen carriers between each cycle.

Even though the hydrogen content of formate (up to 2.85 wt %) is lower than that of FA (4.35 wt %), the presented concepts have the inherent advantages of easy transport and handling of the solid (bi)carbonate and formate salts compared to the well-known carbon dioxide/formic acid couple (including our previous work^67,78^). Both the hydrogen acceptor and donor are nontoxic, nonvolatile, noncorrosive, and nonacidic and show high solubility in water.^88^ While the reported study paves the way for building up a new H_2_ storage-release method, for larger scale applications, it is desirable to improve the catalytic efficiency even if an Earth abundant metal-based catalyst is applied.

## Supporting Information Available

Materials and methods, additional tables, and characterization data (PDF)
