# Supplementary material for: Manganese Promoted (Bi)carbonate Hydrogenation and Formate Dehydrogenation: Toward a Circular Carbon and Hydrogen Economy
Source: ACS Cent Sci. 2022 Oct 19;8(10):1457–63. doi: 10.1021/acscentsci.2c00723 (PMC9615124; doi:10.1021/acscentsci.2c00723)
Supplement: Supplementary file 1 — oc2c00723_si_002.pdf [file oc2c00723_si_002.pdf]

Supporting Information

Manganese promoted (bi)carbonate hydrogenation and formate dehydrogenation: Towards a circular carbon and hydrogen economy

Duo Wei,[†,‖] Xinzhe Shi,[†,‖] Peter Sponholz,[‡] Henrik Junge,[†,*] and Matthias Beller[†,*]

[†] Leibniz-Institut für Katalyse e.V., Albert-Einstein-Str. 29a, 18059 Rostock, Germany

[‡] APEX Energy Teterow GmbH, Hans-Adam-Allee 1, 18299 Rostock-Laage, Germany

[‖]D.W. and X.S. contributed equally to the work

Email: henrik.junge@catalysis.de; matthias.beller@catalysis.de

Table of contents

Materials and methods 2

Standard procedure for catalytic hydrogenation of potassium bicarbonate 3

Standard procedure for catalytic hydrogenation of potassium carbonate 7

Standard procedure for catalytic dehydrogenation of potassium formate 13

Standard procedure for H_2 storage-release cycles starting from formate salts/Lys 21

Standard procedure for H_2 storage-release cycles starting from carbonate salts/Glu 24

References 29

Materials and methods

Unless otherwise stated, all reactions were conducted under an argon atmosphere and all reagents were purchased from commercial suppliers and used without further purification, including L-lysine (fluorochem, 97%), L-arginine (Alfa Aesar, >98%), L-glutamic acid (TCI, >99%), L-aspartic acid (Sigma-Aldrich, >99%), L-histidine (Sigma-Aldrich, >99%), glycine (Sigma-Aldrich, >99%), succinic acid (Alfa Aesar, >99%), propionic acid (Sigma-Aldrich, ≥99.5%), sodium bicarbonate (Merck, >99%), potassium bicarbonate (Sigma-Aldrich, ≥99.5%), cesium bicarbonate (Alfa Aesar, >99%), ammonium bicarbonate (Alfa Aesar, 98%), lithium carbonate (thermo scientific, 99%), sodium carbonate (Acros Organics, 99,8%), potassium carbonates (Sigma-Aldrich, >99%), rubidium carbonate (Strem, ≥99.8%), cesium carbonate (Sigma-Aldrich, 99%), ammonium carbonate (Fisher Scientific, ≥30%), magnesium carbonate (thermo scientific, >99%), calcium carbonate (fluorochem, 99%), barium carbonate (Sigma-Aldrich, >99%), lithium formate monohydrate (Acros Organics, 98%), sodium formate (Acros Organics, 99%), potassium formate (Sigma-Aldrich, 99%), cesium formate (TCI, >98%), ammonium formate (thermo scientific, 97%), Magnesium formate dihydrate (Fluka, ≥98%), calcium formate (Sigma-Aldrich, ≥99%), deuterium oxide (Deutero, D-99.9%), H_2 (Air Liquide, grade 5.0) were used without further purification. Organic solvents were collected from an SPS machine and stored under argon with molecular sieve. THF without stabilizer and deionized (DI) water were used for hydrogenation and dehydrogenation reactions. Organometallic complexes **Mn-1**,[1-2] **Mn-2**,[3] **Mn-3**,[3] **Mn-4**,[3] **Mn-5**,[4] **Mn-6**,[5] **Mn-7**,[6] **Mn-8**,[7] **Fe-1**,[8-9] **Co-1**,[10] were synthesized according to the corresponding publications and stored under argon with light exclusion. No unexpected or unusually high safety hazards were encountered.

^{1}H and $^{13}C\{^{1}H\}$ spectroscopy was recorded using Bruker AV 300 MHz and Bruker AV 400 MHz spectrometers. All NMR data in the experimental section are expressed as chemical shift in parts per million (ppm). ^{1}H and $^{13}C\{^{1}H\}$ chemical shifts were determined relative to the internal standard DMF at 7.92 ppm and 165.53 ppm in D_2O, respectively. NMR spectra were interpreted and processed using MestReNova (version 14.0.1-23559). Gas chromatography (Agilent Technologies 7890A GC system, Carboxen / TCD) was used to analyze the content of the gas phase with a CO quantification limit of 10 ppm.

Standard procedure for catalytic hydrogenation of potassium bicarbonate

Potassium bicarbonate ($KHCO_3$, 10 mmol), **Mn-2** (0.1 mg, 0.18 μmol), THF (5 mL) and H_2O (5 mL) were added to a 100 mL autoclave equipped with a magnetic stir bar. After pressurizing the reactor with H_2 (60 bar), the reaction mixture was heated and stirred on a pre-heated oil bath for 12 h. Then the reactor was cooled to r.t. and the inside pressure was carefully released. A biphasic reaction mixture was obtained containing a transparent organic upper layer and an aqueous yellow lower layer. Addition of DI water (*ca.* 3 mL) to the above mentioned mixture resulted in a homogeneous solution.[11] DMF (500 μL, 6.48 mmol) was added as an NMR internal standard to the reaction mixture, which was then analyzed by ^{1}H NMR with *ca.* 0.1 mL D_2O to lock the signals.[12] Yield of formate is calculated by (mmol formate)/(mmol $KHCO_3$)×100%. All experiments were performed at least twice; average values are shown (St. Dev.<10%).

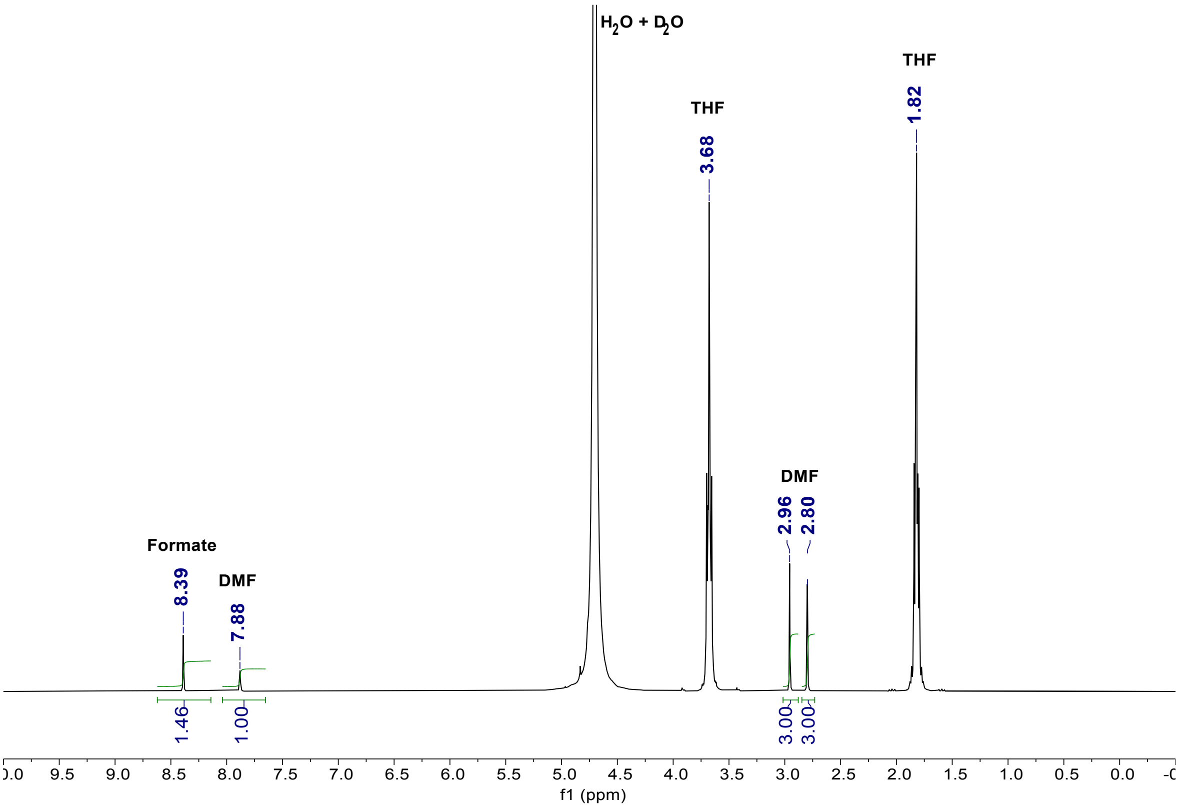

Figure S1. Typical ^{1}H NMR in D_2O after hydrogenation of $KHCO_3$ to formate catalyzed by **Mn-2**. DMF (500 µL, 6.48 mmol) as internal standard.

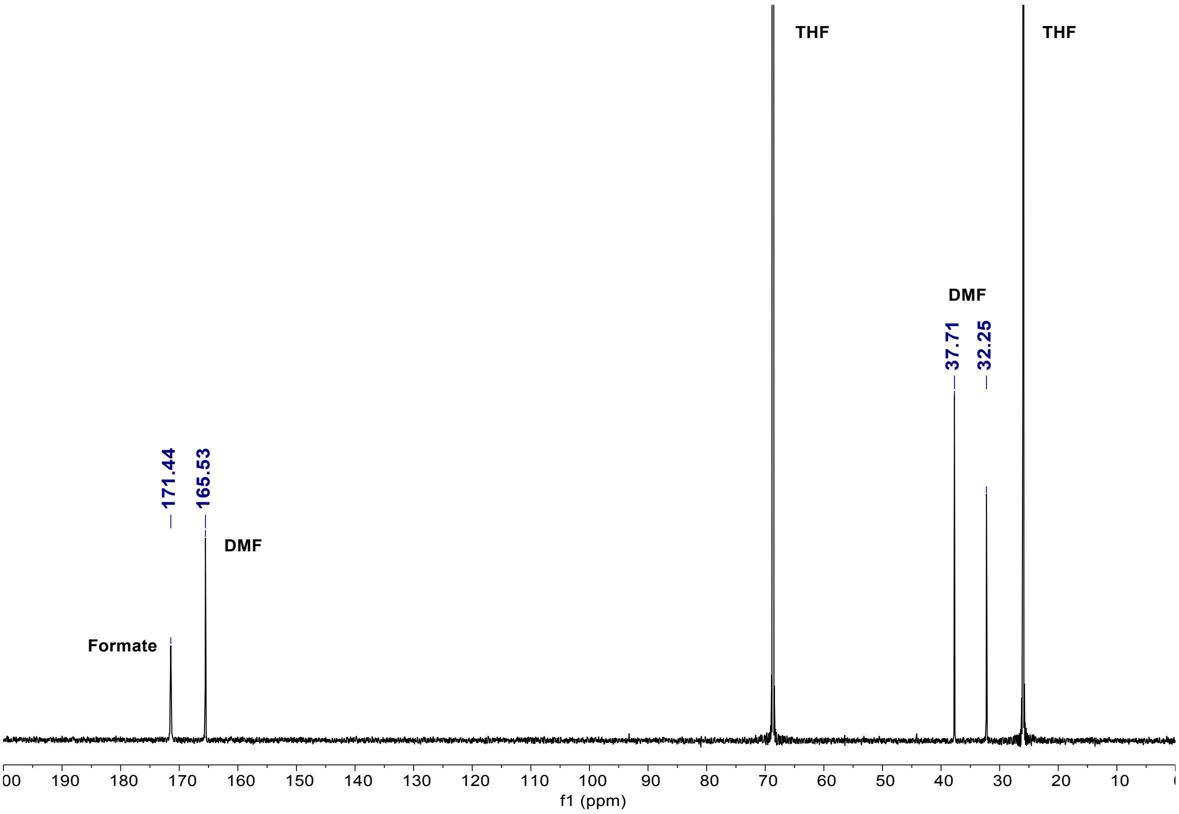

Figure S2. Typical $^{13}C\{^{1}H\}$ NMR in D_2O after hydrogenation of $KHCO_3$ to formate catalyzed by **Mn-2**. DMF (500 µL, 6.48 mmol) as internal standard.

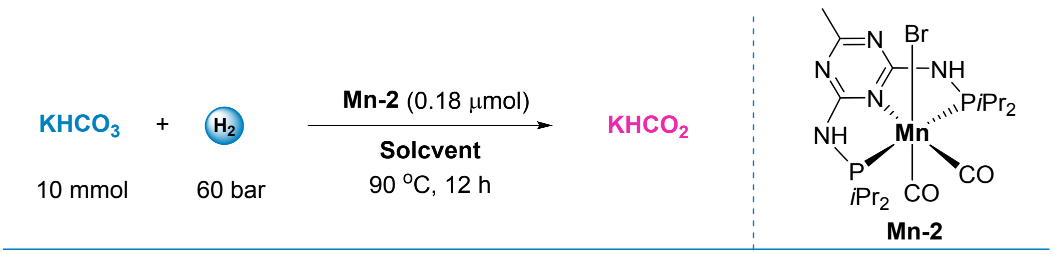

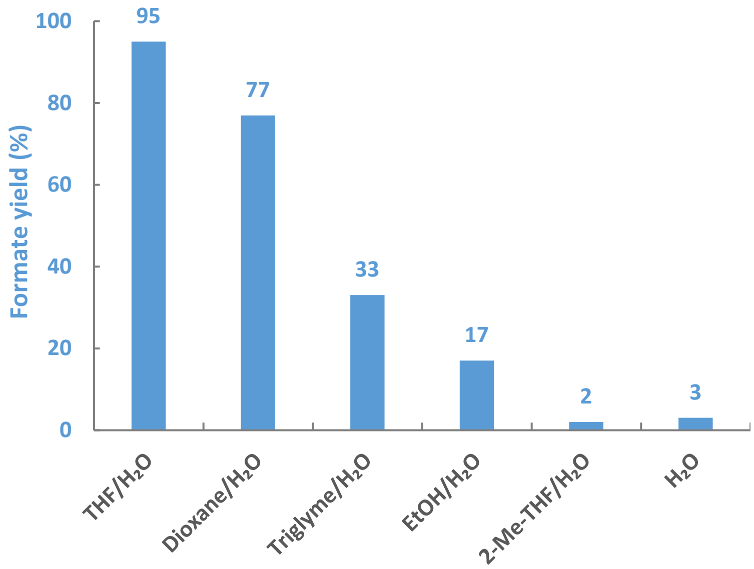

Figure S3. Hydrogenation of potassium bicarbonate catalyzed by **Mn-2**: solvent screening. General conditions: $KHCO_3$ (10 mmol), **Mn-2** (0.1 mg, 0.18 μmol), H_2O/organic solvent (5/5 mL), H_2 (60 bar), 90 °C, 12 h. Amount of formate is determined by ^1H NMR with DMF (500 μL, 6.48 mmol) as internal standard. Yield of formate is calculated by (mmol formate)/(mmol $KHCO_3$)×100%. All experiments were performed at least twice; average values are shown (St. Dev.<10%).

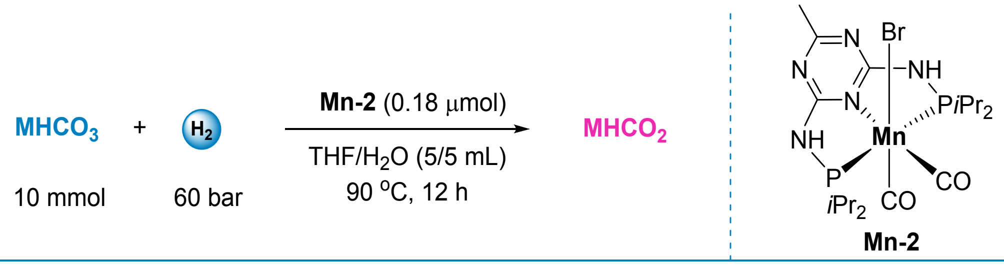

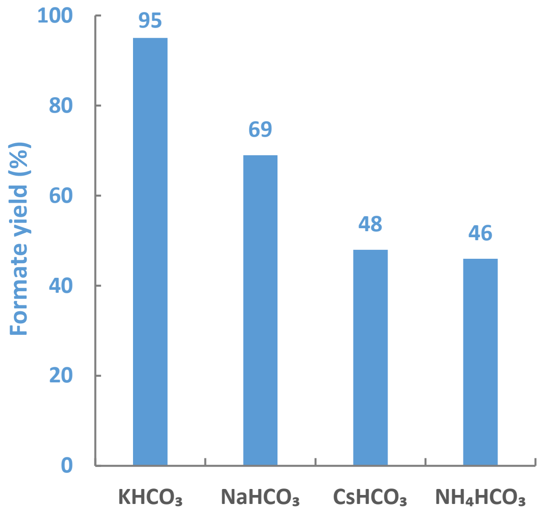

Figure S4. Hydrogenation of various bicarbonate salts catalyzed by **Mn-2**. General conditions: bicarbonate salt (10 mmol), **Mn-2** (0.1 mg, 0.18 μmol), H_2O/THF (5/5 mL), H_2 (60 bar), 90 °C, 12 h. Amount of formate is determined by ^{1}H NMR with DMF (500 μL, 6.48 mmol) as internal standard. Yield of formate is calculated by (mmol formate)/(mmol bicarbonate salt)×100%. All experiments were performed at least twice; average values are shown (St. Dev.<10%).

Standard procedure for catalytic hydrogenation of potassium carbonate

Potassium carbonate (K_2CO_3, 10 mmol), **Mn-2** (0.1 mg, 0.18 μmol), glutamic acid (10 mmol), THF (5 mL) and H_2O (5 mL) were added to a 100 mL autoclave equipped with a magnetic stir bar. After pressurizing the reactor with H_2 (60 bar), the reaction mixture was heated and stirred on a pre-heated oil bath for 12 h. Then the reactor was cooled to r.t. and the inside pressure was carefully released. A biphasic reaction mixture was obtained containing a transparent organic upper layer and an aqueous yellow lower layer. Addition of DI water (*ca.* 3 mL) to the above mentioned mixture resulted in a homogeneous solution.[11] DMF (500 μL, 6.48 mmol) was added as an internal standard to the reaction mixture. The reaction mixture was then analyzed by ^{1}H NMR with *ca.* 0.1 mL D_2O to lock the signals.[12] Yield of formate is calculated by (mmol formate)/(mmol $KHCO_3$)×100%.

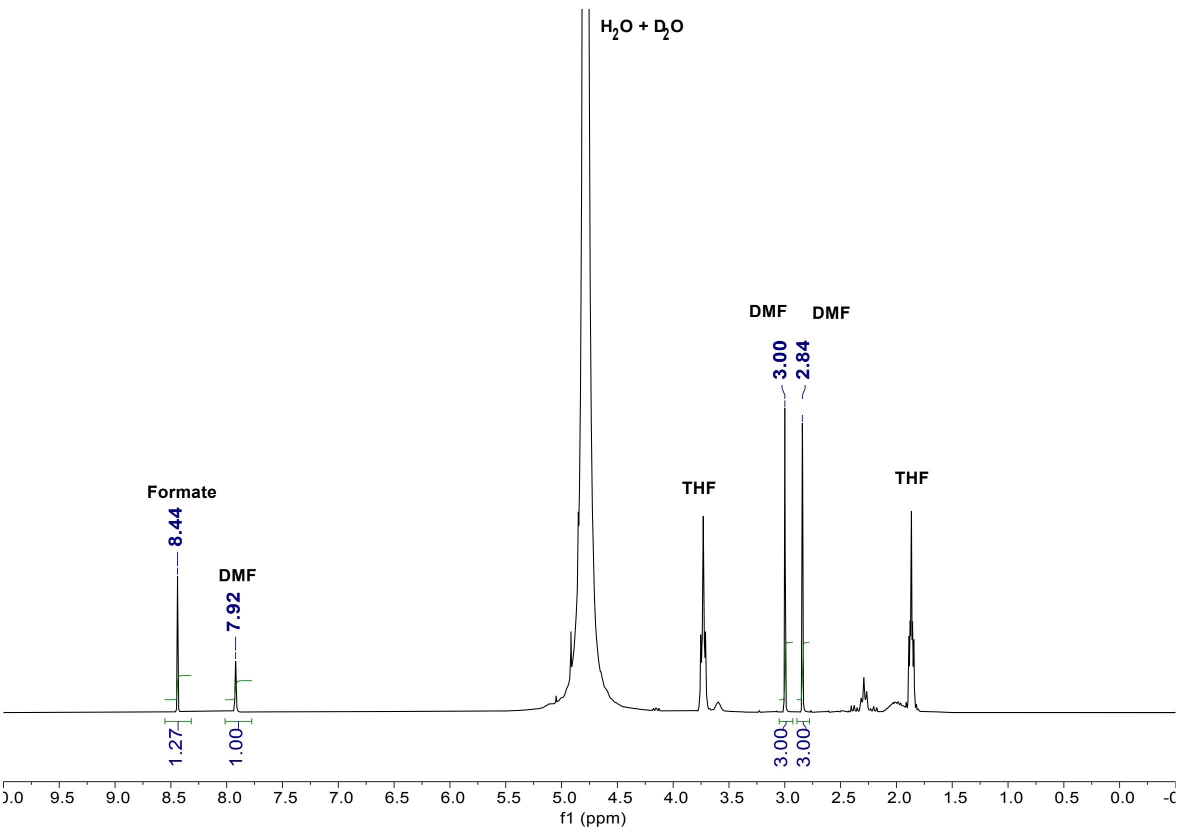

Figure S5. Typical ^{1}H NMR in D_2O after hydrogenation of K_2CO_3 to formate in the presence of glutamic acid (Glu) catalyzed by **Mn-2**. DMF (500 µL, 6.48 mmol) as internal standard.

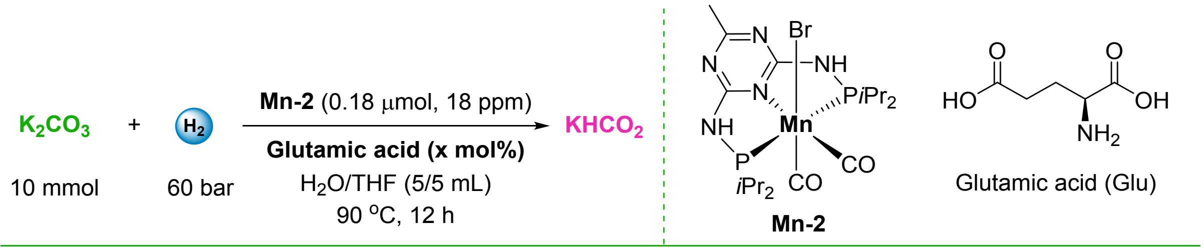

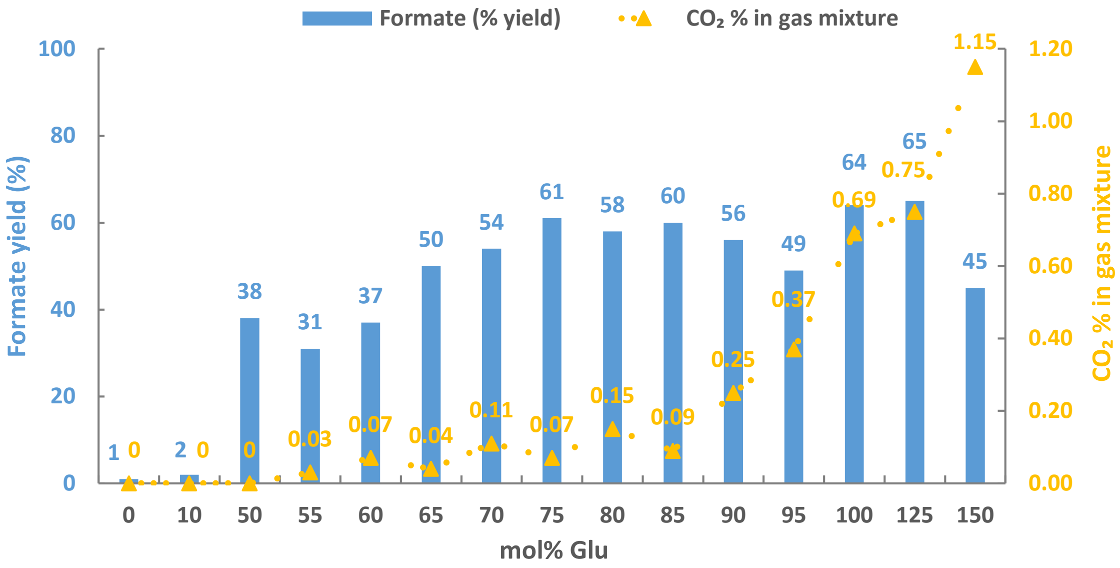

Figure S6. Hydrogenation of potassium carbonates catalyzed by **Mn-2** in the presence of various amount of glutamic acid. General conditions: potassium carbonate (K_2CO_3, 10 mmol), **Mn-2** (0.1 mg, 0.18 μmol), glutamic acid (0-150 mol%), H_2O/THF (5/5 mL), H_2 (60 bar), 90 °C, 12 h. Amount of formate is determined by ^{1}H NMR with DMF (500 μL, 6.48 mmol) as internal standard. Yield of formate is calculated by (mmol formate)/(mmol potassium carbonate)×100%. All experiments were performed at least twice; average values are shown (St. Dev.<10%). The dotted lines serve as guides to the eye.

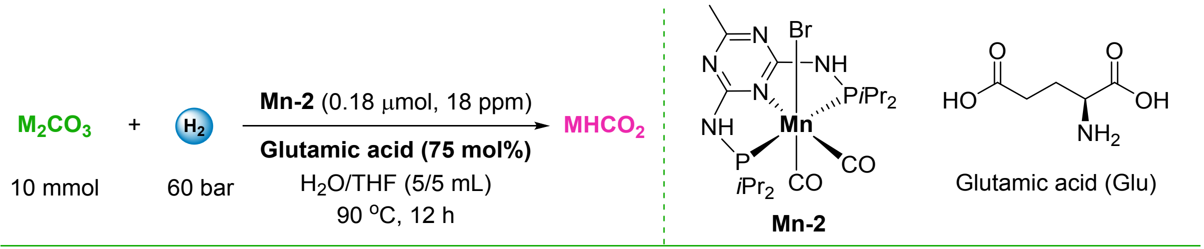

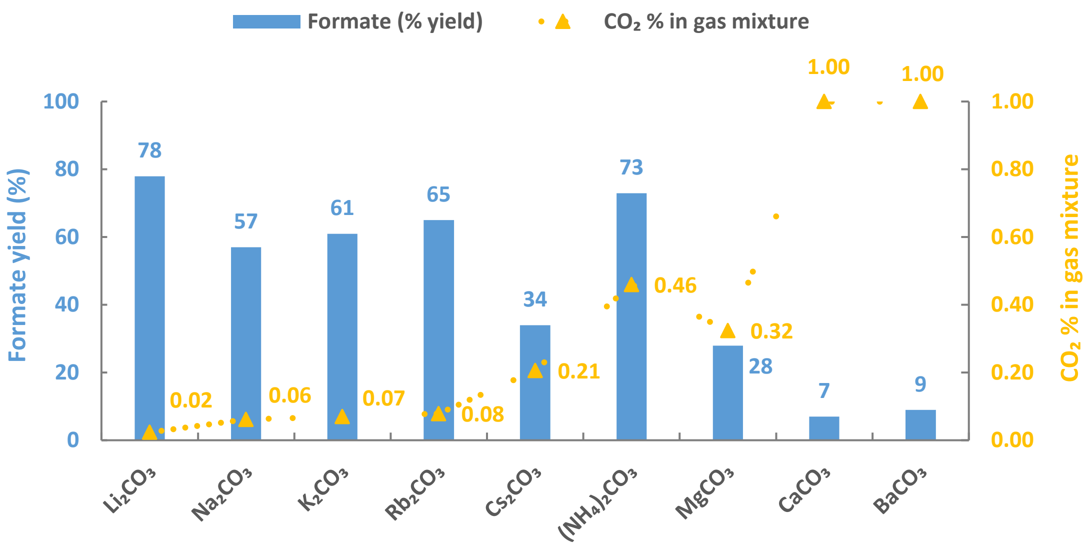

Figure S7. Mn-2 catalyzed hydrogenation of various carbonate salt in the presence of glutamic acid. General conditions: carbonate salt (10 mmol), **Mn-2** (0.1 mg, 0.18 μmol), H_2O/THF (5/5 mL), H_2 (60 bar), glutamic acid (7.5 mmol), 90 °C, 12 h. Amount of formate is determined by ^1H NMR with DMF (500 μL, 6.48 mmol) as internal standard. Yield of formate is calculated by (mmol formate)/(mmol carbonate salt)×100%. All experiments were performed at least twice; average values are shown (St. Dev.<10%). The dotted lines serve as guides to the eye.

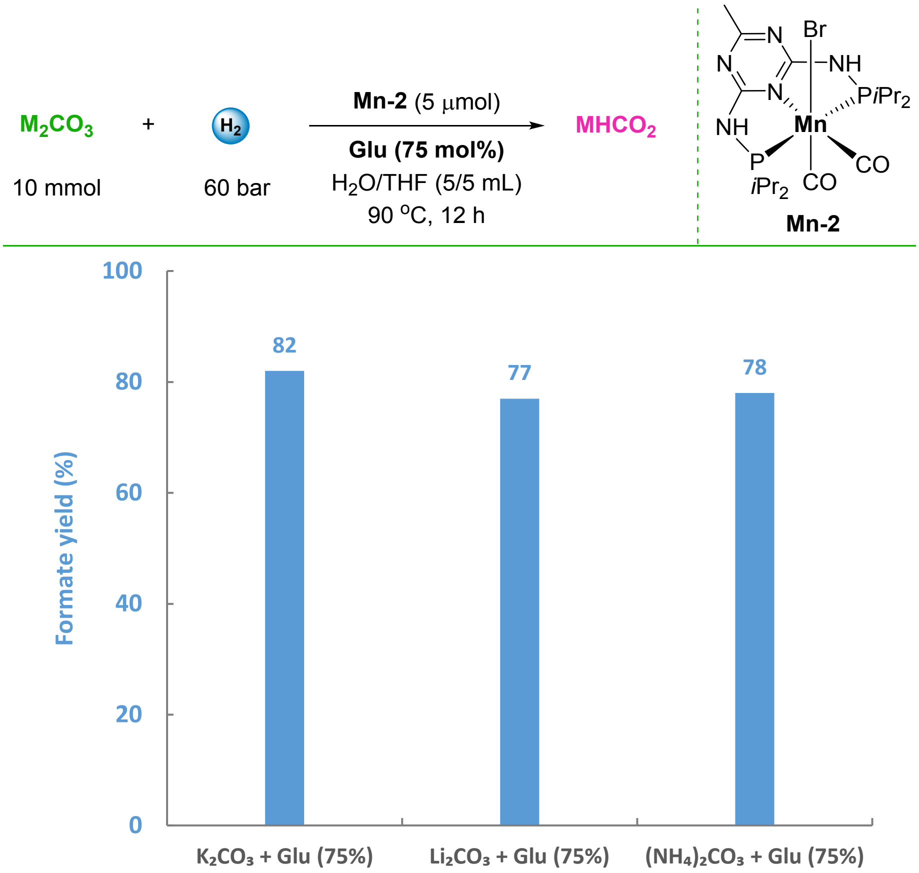

Figure S8. Hydrogenation of various carbonate salts catalyzed by 5 μmol **Mn-2** in the presence of Glu. General conditions: carbonate salt (10 mmol), **Mn-2** (2.7 mg, 5 μmol), H_2O/THF (5/5 mL), H_2 (60 bar), Glu (75 mol%), 90 °C, 12 h. Amount of formate is determined by ^{1}H NMR with DMF (500 μL, 6.48 mmol) as internal standard. Yield of formate is calculated by (mmol formate)/(mmol carbonate salt)×100%. All experiments were performed at least twice; average values are shown (St. Dev.<10%).

Table S1. Effect of different acids on the formation of bicarbonate, CO_2, and carbamate starting from potassium carbonate.[a]

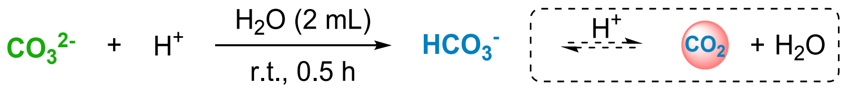

Entry	Acid (H^+)	Bicarbonate	CO_2	Carbamate
1	Propionic acid	71%	8%	0
2	Succinic acid	3%	95%	0
3	Glutamic acid	51%	16%	26%

[a] Conditions: K_2CO_3 (5.0 mmol), acid (5.0 mmol), H_2O (2 mL), r.t., 0.5 h. Yield of bicarbonate and carbamate were determined by ^{13}C NMR-quant. Yield of CO_2 was determined by manual burette.

Standard procedure for catalytic dehydrogenation of potassium formate

Potassium formate ($KHCO_2$, 5.0 mmol), catalyst, THF (5 mL) and H_2O (5 mL) were added to a 50 mL pressure tube equipped with a magnetic stir bar. The reaction mixture was then heated and stirred on a pre-heated oil bath for 12 h. The mixture was cooled to r.t. and the inside pressure was released carefully to the manual burettes. A 5 mL degassed syringe was used to obtain a gas sample analyzed by gas chromatography (GC). CO is not detectable in all cases (below the CO quantification limit of 10 ppm). Yield of H_2 is calculated by (mmol H_2)/(mmol $KHCO_2$)×100%. TON of H_2 is calculated by (mmol H_2)/(mmol catalyst).

Calculation of the hydrogen volume, mole, yield, and the TON. The gas evolution was corrected with the blank volume (18 mL) which corresponds to the gas evolution of the same reaction without any catalyst.

H_2 volume V_{H_2} and CO_2 volume V_{CO_2} are calculated with the following equation:

$$V_{H_2} = (V_{obs} - V_{blank}) \times \frac{\%V_{H_2}}{\%V_{H_2} + \%V_{CO_2}}$$

$$V_{CO_2} = (V_{obs} - V_{blank}) \times \frac{\%V_{CO_2}}{\%V_{H_2} + \%V_{CO_2}}$$

Moles of H_2 n_{H_2} and moles of CO_2 n_{CO_2} are calculated with the following equation:

$$n_{H_2} = \frac{V_{H_2}}{V_{m_{H_2}(25^oC)}}$$

$$n_{CO_2} = \frac{V_{CO_2}}{V_{m_{CO_2}(25^oC)}}$$

Hydrogen yield Y_{H_2} is calculated with the following equation:

$$Y_{H_2} = \frac{n_{H_2}}{n_{FA}} \times 100\%$$

The turnover number (TON) of H_2 is calculated with the following equation:

$$TON = \frac{n_{H_2}}{n_{cat}}$$

Where:

- V_{obs} is the gas evolution volume of catalytic reaction measured in the manual burettes.

- V_{blank} is the gas evolution volume of the blank reaction measured in the manual burettes.
- $\%V_{H_2}$ and $\%V_{CO_2}$ are the volume ratios of H_2 and CO_2, respectively, determined by GC.
- n_{FA} and n_{cat} are the moles of FA and catalyst, respectively.
- $V_{m_{H_2\,(25^oC)}}$ and $V_{m_{CO_2\,(25^oC)}}$ are the molar volumes of H_2 and CO_2 at room temperature (25 °C), respectively, calculated with the Van Der Waals equation, see table below:

Calculation of H_2 molar volume $V_{m_{H_2\,(25^oC)}}$:	Calculation of CO_2 molar volume $V_{m_{CO_2\,(25^oC)}}$:
$V_{m_{H_2\,(25^oC)}} = \frac{R \times T}{p} + b - \frac{a}{R \times T} = 24.48$<br>Where:<br>• R = 8.3145 $m^3 \cdot Pa \cdot mol^{-1} \cdot K^{-1}$<br>• T = 273.15 + room temperature (°C) K<br>• P = 101325 Pa<br>• a = 24.9×10^{-3} $Pa \cdot m^6 \cdot mol^{-2}$<br>• b = 26.7×10^{-6} $m^3 \cdot mol^{-1}$	$V_{m_{CO_2\,(25^oC)}} = \frac{R \times T}{p} + b - \frac{a}{R \times T} = 24.36$<br>Where:<br>• R = 8.3145 $m^3 \cdot Pa \cdot mol^{-1} \cdot K^{-1}$<br>• T = 273.15 + room temperature (°C) K<br>• P = 101325 Pa<br>• a = 36.5×10^{-2} $Pa \cdot m^6 \cdot mol^{-2}$<br>• b = 42.7×10^{-6} $m^3 \cdot mol^{-1}$

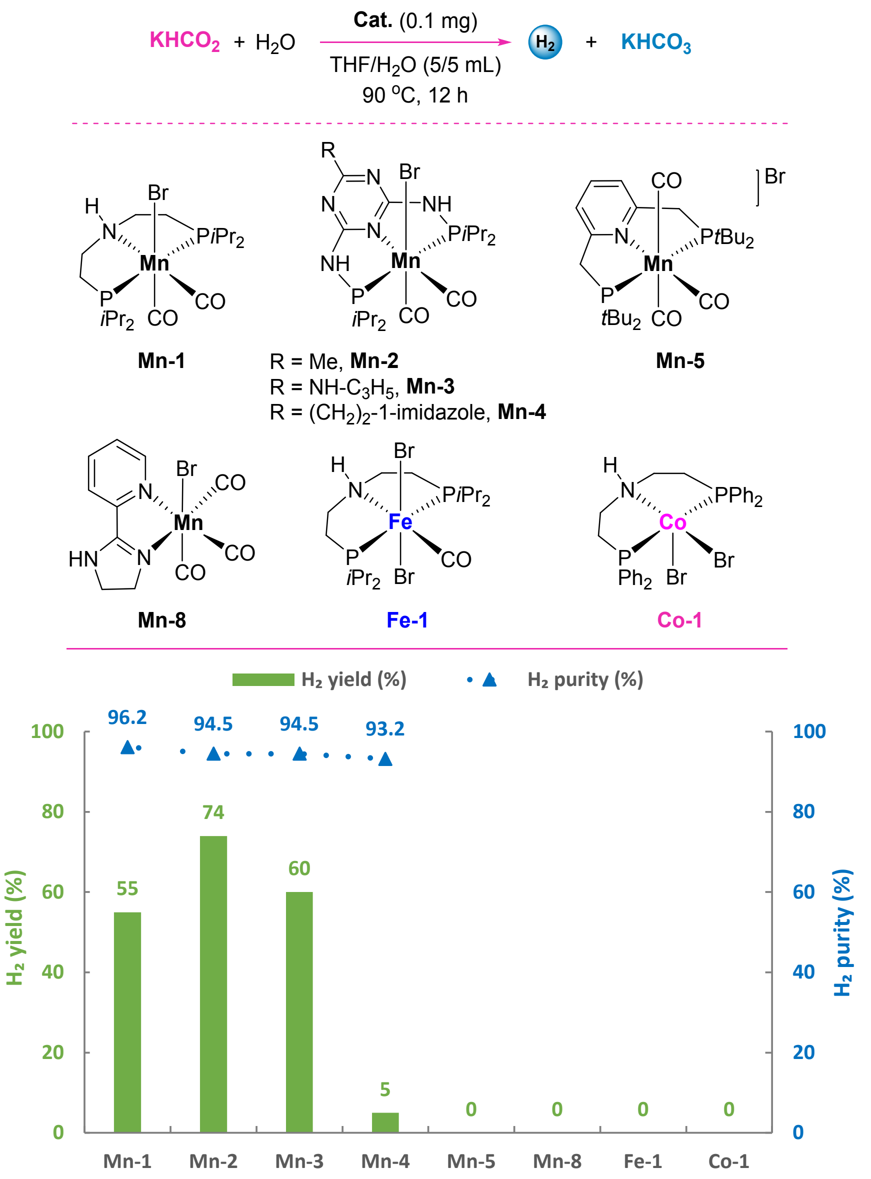

Figure S9. Catalytic dehydrogenation of potassium formate: catalysts screening in the absence of additives. General conditions: $KHCO_2$ (5.0 mmol), catalyst (0.1 mg), H_2O/THF (5/5 mL), 90 °C, 12 h. Volume and content of the gas phase from manual burettes were analyzed by gas chromatography (GC) after correction by the blank volume. CO is not detectable in all cases (below the CO quantification limit of 10 ppm). Yield of H_2 is calculated by (mmol H_2)/(mmol $KHCO_2$)×100%. The dotted lines serve as guides to the eye.

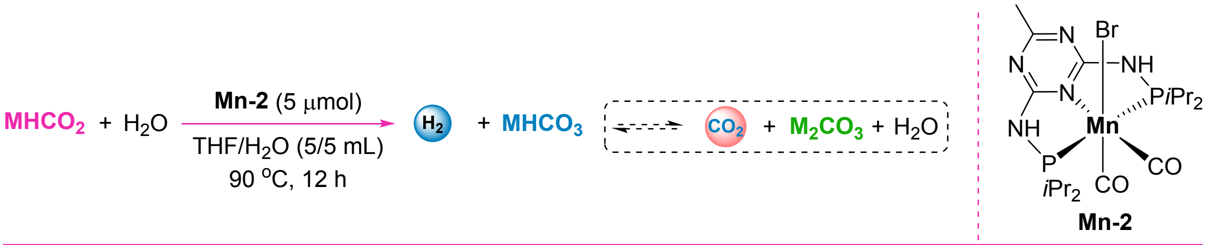

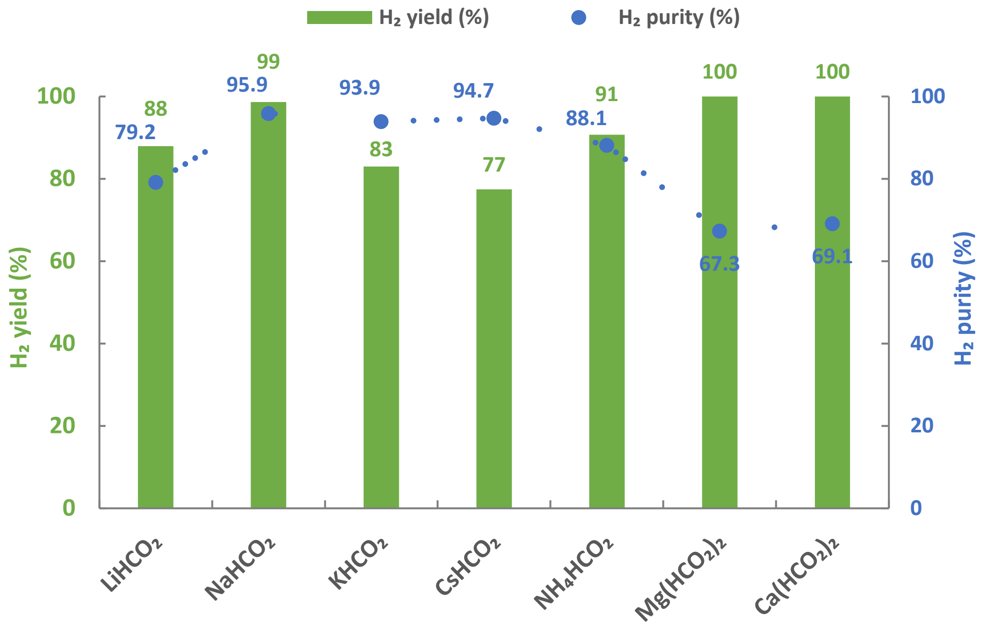

Figure S10. Catalytic dehydrogenation of various formate salts in the absence of additives. General conditions: formate salt (5.0 mmol), **Mn-2** (2.7 mg, 5 μmol), H_2O/THF (5/5 mL), 90 °C, 12 h. Volume and content of the gas phase from manual burettes were analyzed by gas chromatography (GC) after correction by the blank volume. CO is not detectable in all cases (below the CO quantification limit of 10 ppm). Yield of H_2 is calculated by (mmol H_2)/(mmol $KHCO_2$)×100%. The dotted lines serve as guides to the eye.

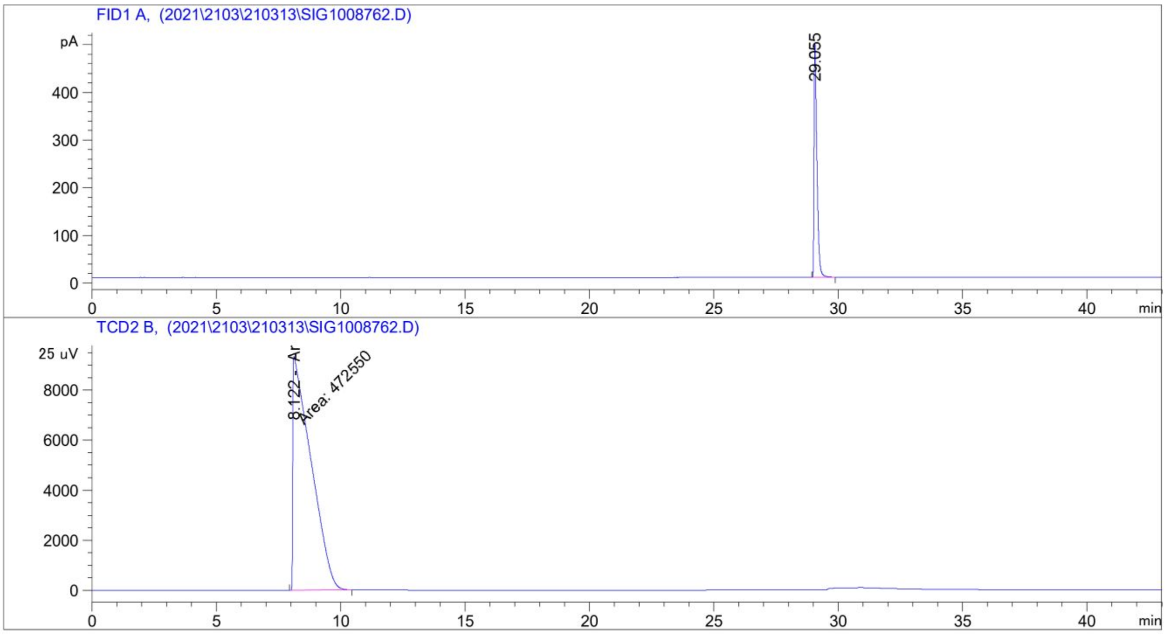

===
External Standard Report
===

Sorted By : Retention Time
Calib. Data Modified : 4/6/2022 12:33:11 PM
Multiplier : 1.0000
Dilution : 1.0000
Use Multiplier & Dilution Factor with ISTDs

Signal 1: FID1 A,
Signal 2: TCD2 B,

RetTime [min]	Sig	Type	Area	Amt/Area	Amount [vol%]	Grp	Name
2.846	2		-	-	-		H2
8.122	2	MM	4.72550e5	2.16753e-4	102.42691		Ar
12.105	2		-	-	-		CO
21.000	2		-	-	-		CH4
27.935	2		-	-	-		CO2

Figure S11. GC chromatogram of blank reaction for the dehydrogenation of FA. Only argon was reported at retention time 8.122 min.

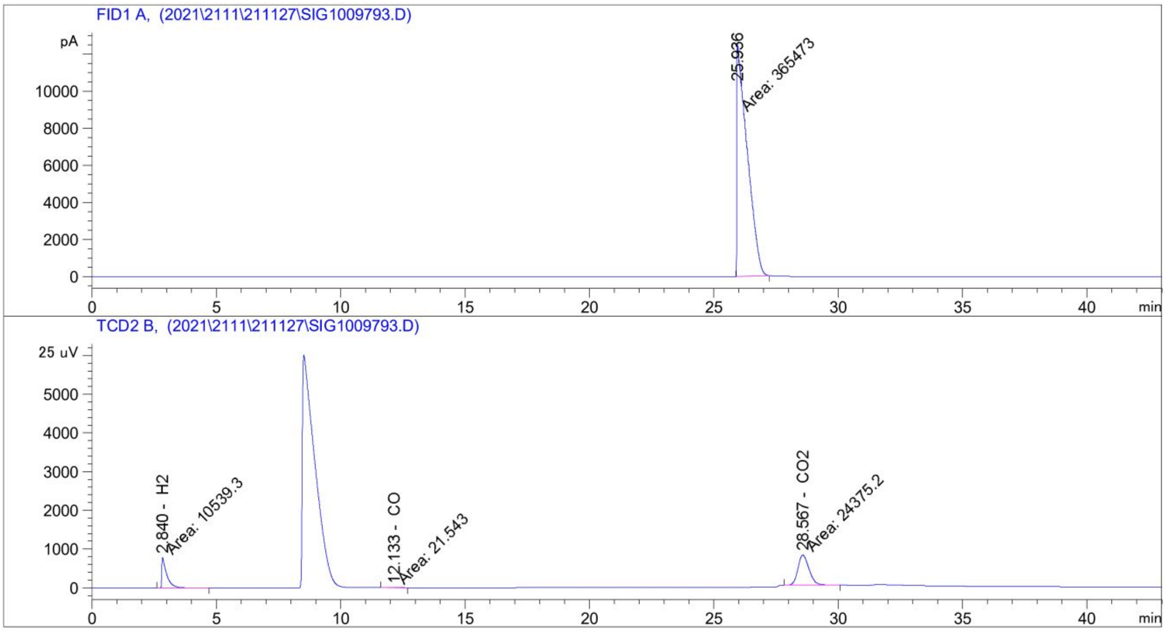


```
=====================================================================
                     External Standard Report
=====================================================================

Sorted By              :      Retention Time
Calib. Data Modified   :      4/6/2022 12:34:52 PM
Multiplier             :      1.0000
Dilution               :      1.0000
Use Multiplier & Dilution Factor with ISTDs

Signal 1: FID1 A,
Signal 2: TCD2 B,

RetTime Sig Type     Area      Amt/Area     Amount    Grp   Name
 [min]                                      [vol%]
-------|--|------|----------|----------|----------|--|---------------
  2.840  2 MM     1.05393e4  4.43418e-3   46.73294     H2
  8.122  2            -          -           -         Ar
 12.133  2 MM       21.54300    0.00000    0.00000     CO
 21.000  2            -          -           -         CH4
 28.567  2 MM     2.43752e4  1.49432e-4    3.64245     CO2
```

Figure S12. Typical GC chromatogram of potassium formate dehydrogenation in the absence of additives.

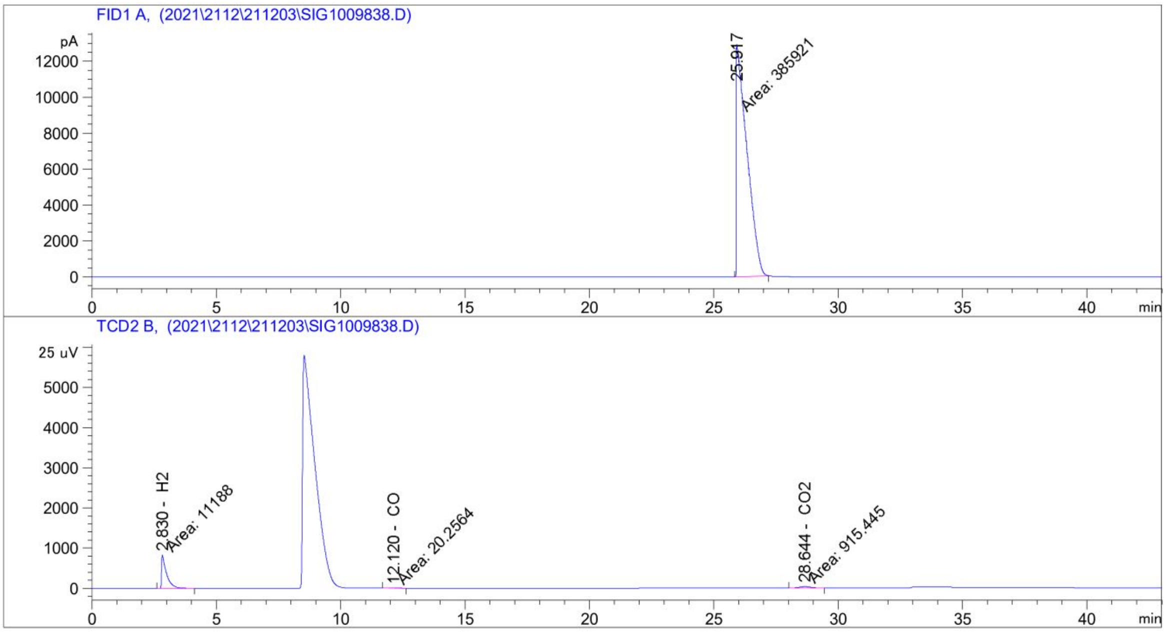

===
External Standard Report
===

Sorted By : Retention Time
Calib. Data Modified : 4/6/2022 12:36:11 PM
Multiplier : 1.0000
Dilution : 1.0000
Use Multiplier & Dilution Factor with ISTDs

Signal 1: FID1 A,
Signal 2: TCD2 B,

RetTime [min]	Sig	Type	Area	Amt/Area	Amount [vol%]	Grp	Name
2.830	2	MM	1.11880e4	4.42156e-3	49.46845		H2
8.122	2		-	-	-		Ar
12.120	2	MM	20.25644	0.00000	0.00000		CO
21.000	2		-	-	-		CH4
28.644	2	MM	915.44489	1.42165e-4	1.30144e-1		CO2

Figure S13. Typical GC chromatogram of potassium formate dehydrogenation in the presence of L-lysine (Lys).

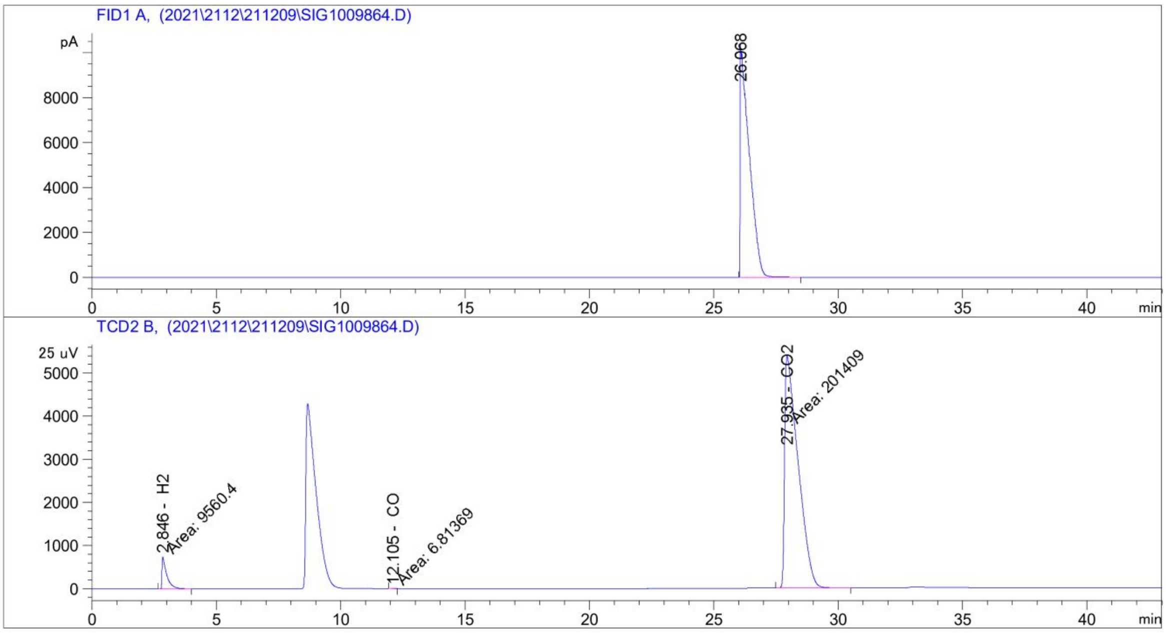


```
=====================================================================
                      External Standard Report
=====================================================================

Sorted By             :      Retention Time
Calib. Data Modified  :      4/6/2022 12:19:07 PM
Multiplier            :      1.0000
Dilution              :      1.0000
Use Multiplier & Dilution Factor with ISTDs

Signal 1: FID1 A,
Signal 2: TCD2 B,

RetTime Sig Type     Area      Amt/Area     Amount    Grp   Name
 [min]                                      [vol%]
-------|--|------|----------|----------|----------|--|---------------
  2.846  2 MM     9560.39941 4.45351e-3   42.57738    H2
  8.050  2           -          -           -         Ar
 12.105  2 MM        6.81369    0.00000    0.00000    CO
 21.000  2           -          -           -         CH4
 27.935  2 MM     2.01409e5  1.52752e-4   30.76563    CO2
```

Figure S14. Typical GC chromatogram of potassium formate dehydrogenation in the presence of L-glutamic acid (Glu).

Standard procedure for H_2 storage-release cycles starting from formate salts/Lys

The H_2 storage-release cycles starts from the dehydrogenation of formate salt (<u>H_2 release</u>): **Mn-2** (5 µmol), formate salt (5.0 mmol), Lys, THF (5 mL) and H_2O (5 mL) were added to a 100 mL autoclave equipped with a magnetic stir bar. The reaction mixture was then heated and stirred on a pre-heated oil bath at 90 °C for 12 h. The reactor was cooled to r.t. and the inside pressure was released carefully to the manual burettes and the content of the gas phase was analyzed by gas chromatography (GC). CO is not detectable in all cases (below the CO quantification limit of 10 ppm). The autoclave was then filled with 60 bar of H_2, heated and stirred on a pre-heated oil bath at 90 °C for 12 h (<u>H_2 storage</u>). After the completion of H_2 storage, the reactor was cooled to r.t. and the inside pressure was released carefully. Then the autoclave was subjected to the <u>H_2 release</u> procedure again. Following such process, the H_2 evolution in the H_2 storage-release cycles were implemented with various formate salts.

Table S2. H_2 evolution yields in the H_2 storage-release cycles applying formate salt (5.0 mmol).[a]

Cycle / Substrate	1st	2nd	3rd	4th	5th	Formate salt[b]
**$LiHCO_2$**	99	75	62	91	20	19
**$NaHCO_2$**	99	78	71	58	48	45
**$KHCO_2$**	96	85	83	80	77	68
**$CsHCO_2$**	99	72	68	46	38	32
**NH_4HCO_2**	99	89	57	32	21	19
**$Ca(HCO_2)_2$**	99	58	48	36	29	21
**$Mg(HCO_2)_2$**	100	68	58	45	39	30

[a] H_2 yields of each cycle are calculated based on the initial loading of formate salt (5 mmol). [b] Formate salt yields after the hydrogenation step of the final cycle, calculated based on the initial loading of formate salt (5 mmol).

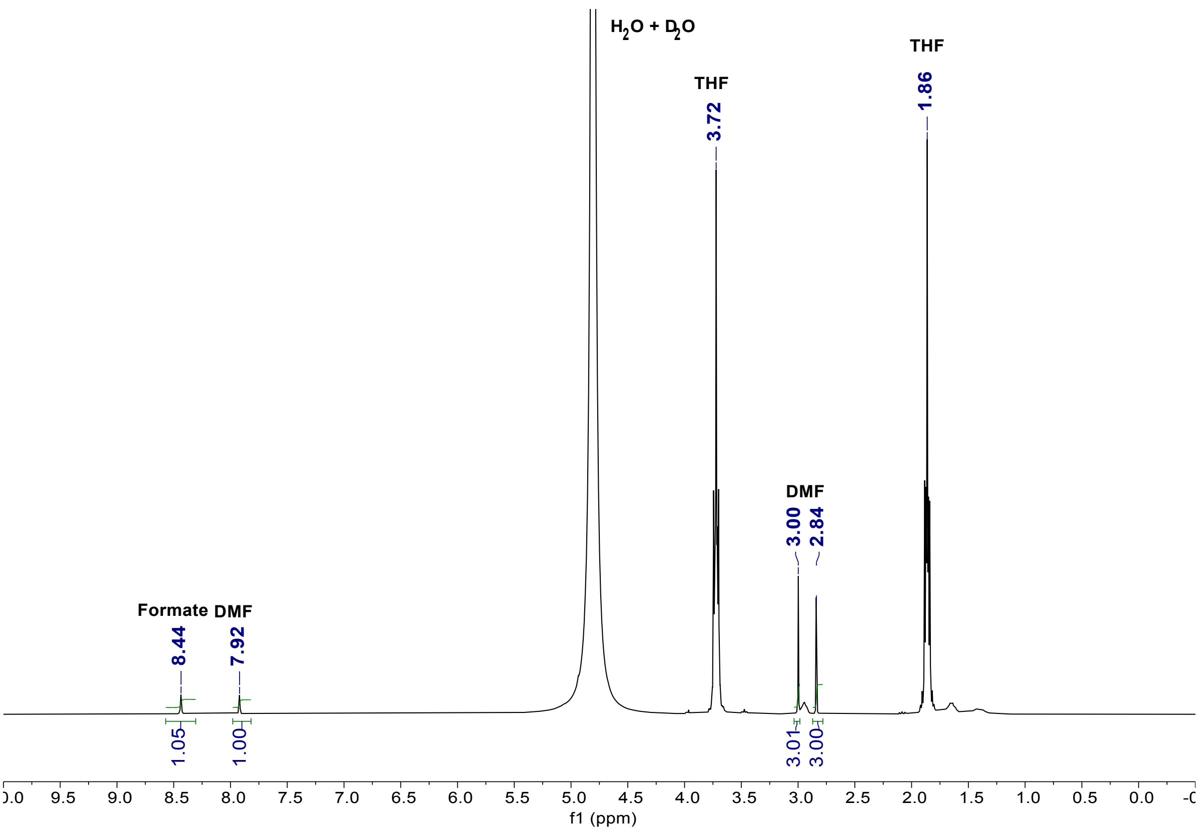

Figure S15. Typical ^{1}H NMR in D_2O of the H_2 cycles starting from formate salt and Lys. DMF (250 µL, 3.24 mmol) as internal standard.

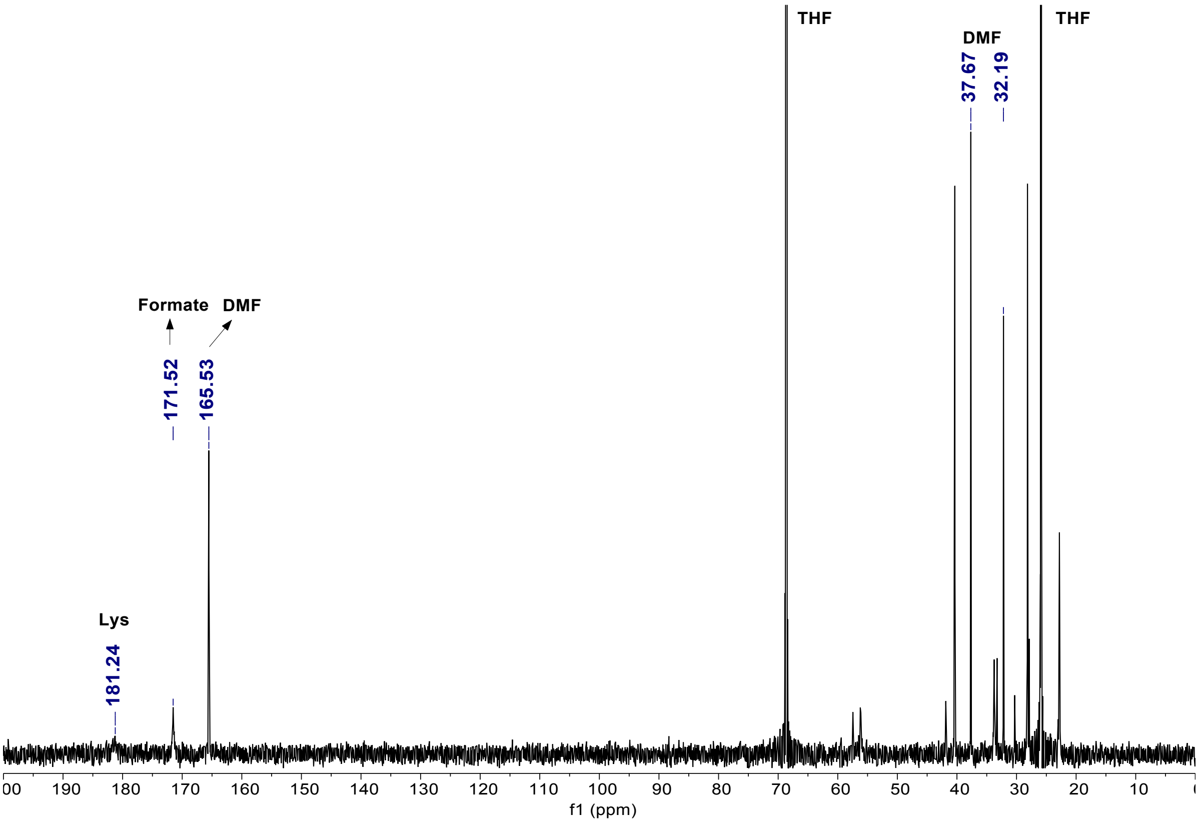

Figure S16. Typical ^{13}C{^{1}H} NMR in D_2O of the H_2 cycles starting from formate salt and Lys. DMF (250 µL, 3.24 mmol) as internal standard.

Standard procedure for H_2 storage-release cycles starting from carbonate salts/Glu

The H_2 storage-release cycles starts from the hydrogenation of carbonate salt (H_2 storage): **Mn-2** (5 μmol), carbonate salt (5.0 mmol), Glu, THF (5 mL) and H_2O (5 mL) were added to a 100 mL autoclave equipped with a magnetic stir bar. After pressurizing the reactor with H_2 (60 bar), the reaction mixture was heated and stirred on a pre-heated oil bath for 12 h. Afterwards, the reactor was cooled to r.t. and the inside pressure was released carefully. The autoclave was then subjected to H_2 release process at 90 °C for 12 h. After the completion of the H_2 release step, the reactor was cooled to r.t. and the inside pressure was released carefully to the manual burettes and the content of the gas phase was analyzed by gas chromatography (GC). CO is not detectable in all cases (below the CO quantification limit of 10 ppm). Then the autoclave was subjected to the H_2 storage procedure again. Following such process, the H_2 evolution in the H_2 storage-release cycles were tested with various carbonate salts.

Table S3. H_2 evolution yields in the H_2 storage-release cycles applying carbonate salt (5.0 mmol).[a]

Cycle / Substrate	1st	2nd	3rd	4th	Formate salt[b]
**K_2CO_3 + Glu [50%]**	34	-	-	-	22
**K_2CO_3 + Glu [75%]**	96	58	-	-	41
**K_2CO_3 + Glu [100%]**	94	87	72	49	36
**Li_2CO_3**	98	89	53	-	40
**Na_2CO_3**	97	99	78	49	33
**Rb_2CO_3**	100	76	66	40	25
**Cs_2CO_3**	100	87	45	-	29
**$(NH_4)_2CO_3$**	93	53	-	-	38
**$MgCO_3$**	67	53	33	-	19
**$CaCO_3$**	28	-	-	-	12
**$BaCO_3$**	33	-	-	-	10

[a] H_2 yields of each cycle are calculated based on the initial loading of carbonate salt (5 mmol). [b] Formate salt yields after the hydrogenation step of the final cycle, calculated based on the initial loading of carbonate salt (5 mmol).

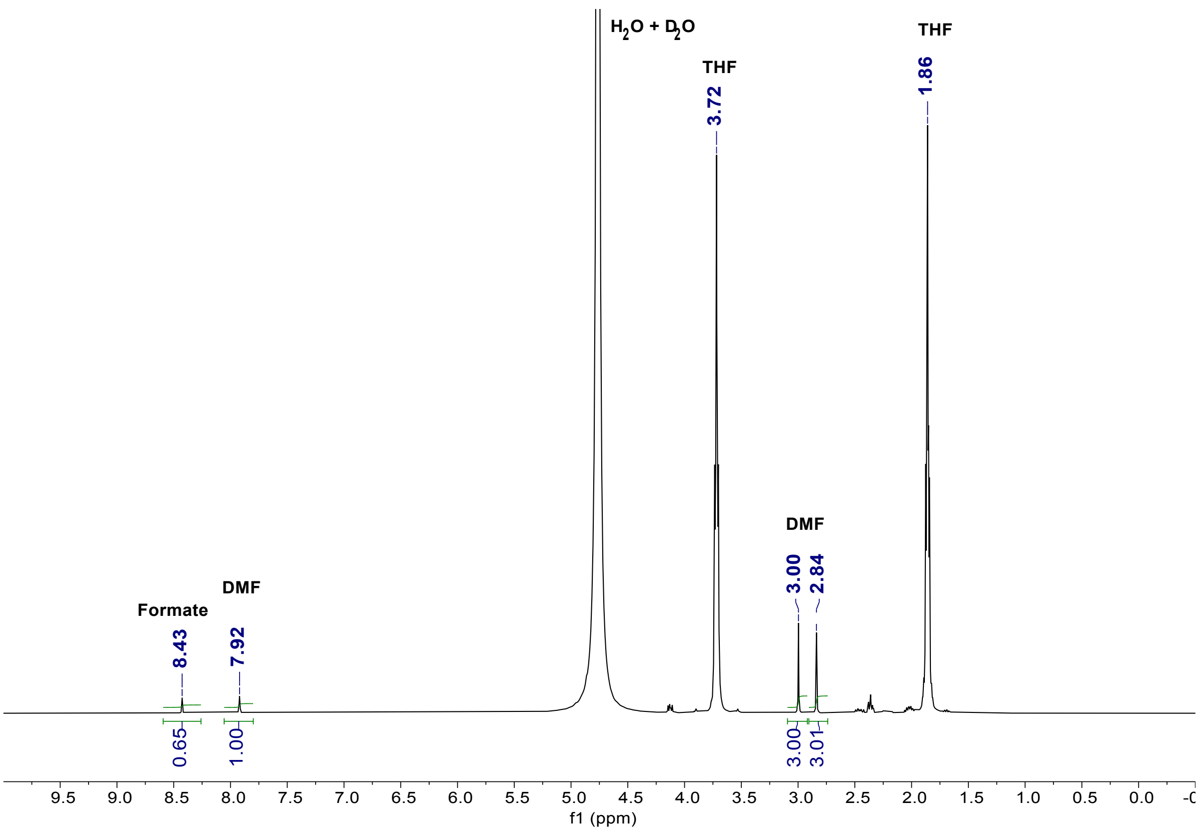

Figure S17. Typical ^{1}H NMR in D_2O of the H_2 cycles applying carbonate salt and Glu. DMF (250 µL, 3.24 mmol) as internal standard.

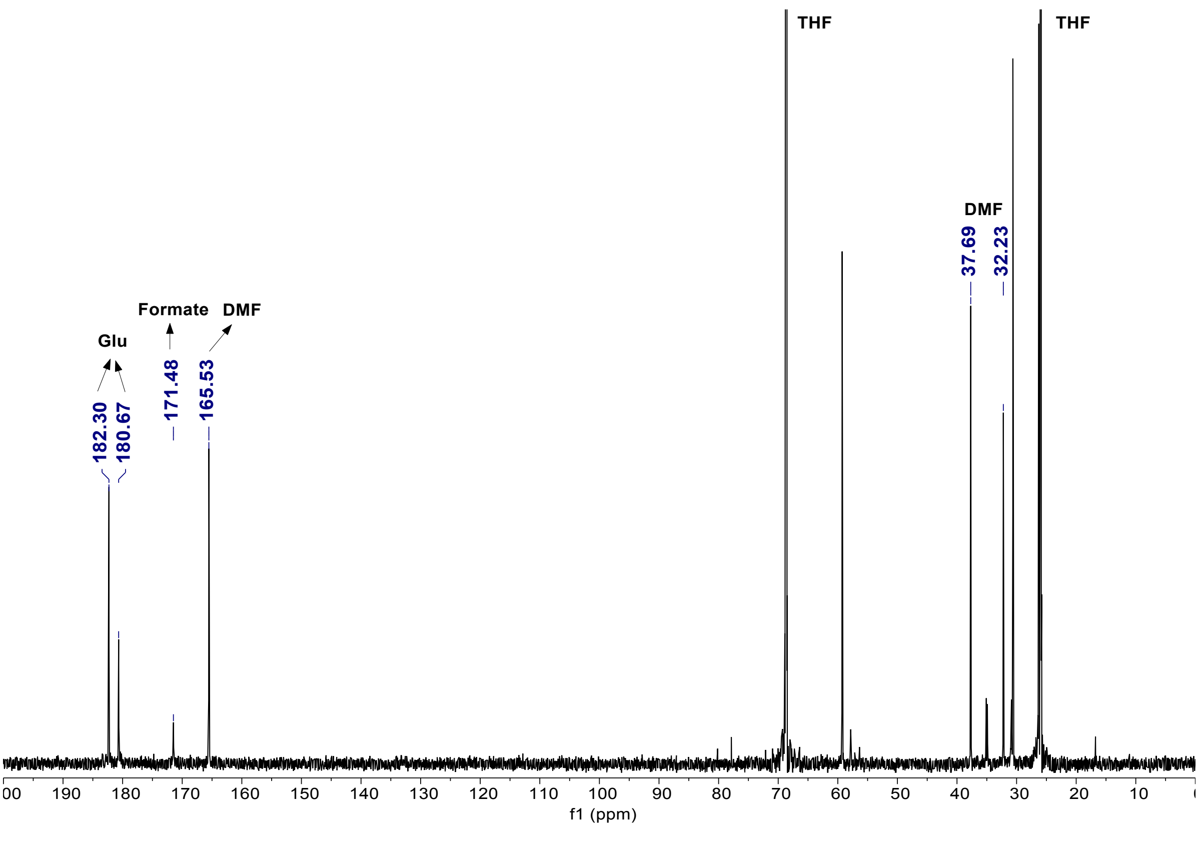

Figure S18. Typical $^{13}C\{^{1}H\}$ NMR in D_2O of the H_2 cycles applying carbonate salt and Glu. DMF (250 µL, 3.24 mmol) as internal standard.

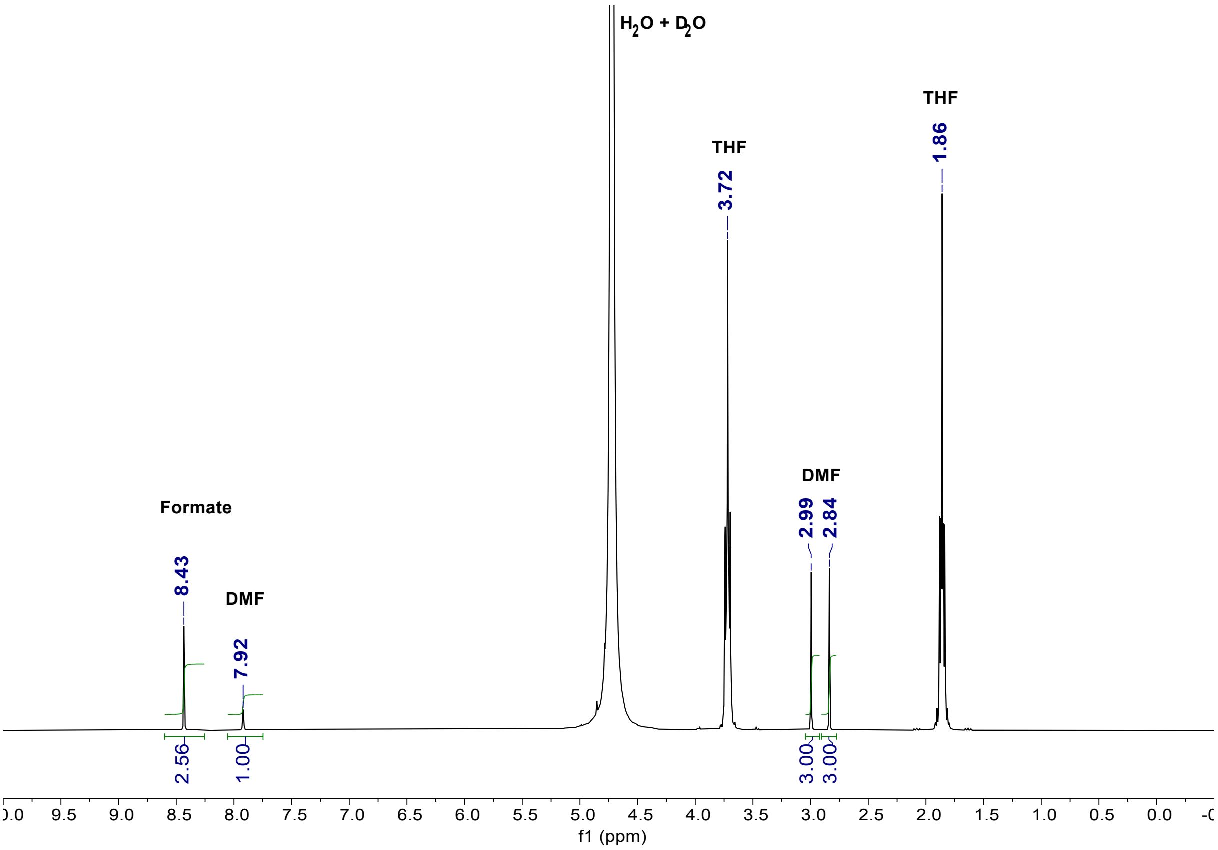

Figure S19. ^{1}H NMR in D_2O of K_2CO_3 (10 mmol) hydrogenation applying H_2/CO_2 (60/10 bar), **Mn-2** (0.18 µmol), H_2O/THF (5/5 mL), 90 °C, 12 h. Internal standard for NMR: DMF (500 µL, 6.48 mmol). Yield of formate is calculated by (mmol formate)/(mmol K_2CO_3 ×2)×100%.

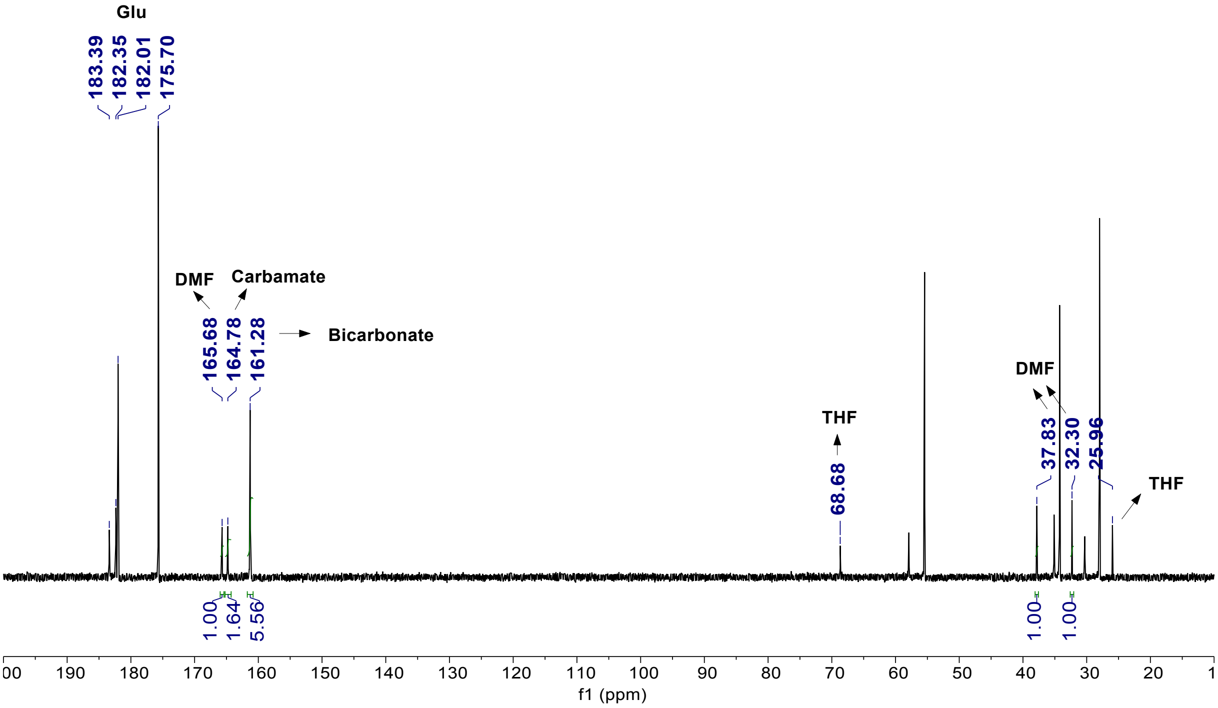

Figure S20. ^{13}C-quant NMR in D_2O after the first H_2 storage-release cycle applying K_2CO_3/Glu (5.0/5.0 mmol), **Mn-2** (5 μmol), H_2O/THF (5/5 mL), 90 °C, 12 h. Internal standard for NMR: DMF (48 μL, 0.625 mmol).

Table S4. Comparison of CO_2 capture ability between K_2CO_3 and Lys.[a]

Entry	Substrate	CO_2 in carbamate [mmol]	CO_2 in bicarbonate [mmol]	total CO_2 captured [mmol][b]
1	K_2CO_3	0	1.8	1.8
2	Lys	2.2	0.7	2.9

[a] Conditions: K_2CO_3 or Lys (5.0 mmol), H_2O (5 mL), CO_2 (2 bar), r.t. (24 °C), 0.5 h. The captured CO_2 amount were calculated by gravimetric analysis. [b] Calculated by (mmol CO_2 in carbamates)+(mmol CO_2 in bicarbonate).

Table S5. Table of pH values.[a]

Compounds	K_2CO_3 (5.0 mmol)	$KHCO_2$ (5.0 mmol)	Glu (5.0 mmol)	K_2CO_3 + Glu (5.0 + 5.0 mmol)	$KHCO_2$ + Glu (5.0 + 5.0 mmol)
**pH**	12.1	8.0	3.2	7.9	4.7

[a] Conditions: compound (5.0 mmol), H_2O (5 mL), r.t. (24 °C). pH values were measured on a laboratory digital pH meter (Mettler-Toledo AG, SevenEasy pH 8603) at r.t. (24 °C).

References

(1) Elangovan, S.; Topf, C.; Fischer, S.; Jiao, H.; Spannenberg, A.; Baumann, W.; Ludwig, R.; Junge, K.; Beller, M. Selective catalytic hydrogenations of nitriles, ketones, and aldehydes by well-defined manganese pincer complexes. *J. Am. Chem. Soc.* **2016,** *138*, 8809–8814.

(2) Elangovan, S.; Neumann, J.; Sortais, J.-B.; Junge, K.; Darcel, C.; Beller, M. Efficient and selective *N*-alkylation of amines with alcohols catalysed by manganese pincer complexes. *Nat. Commun.* **2016,** *7*, 12641.

(3) Kallmeier, F.; Irrgang, T.; Dietel, T.; Kempe, R. Highly Active and Selective Manganese C=O Bond Hydrogenation Catalysts: The Importance of the Multidentate Ligand, the Ancillary Ligands, and the Oxidation State. *Angew. Chem. Int. Ed.* **2016,** *55*, 11806–11809.

(4) Kaithal, A.; Hölscher, M.; Leitner, W. Catalytic Hydrogenation of Cyclic Carbonates using Manganese Complexes. *Angew. Chem. Int. Ed.* **2018,** *57*, 13449–13453.

(5) Papa, V.; Cabrero-Antonino, J. R.; Alberico, E.; Spanneberg, A.; Junge, K.; Junge, H.; Beller, M. Efficient and selective hydrogenation of amides to alcohols and amines using a well-defined manganese–PNN pincer complex. *Chem. Sci.* **2017,** *8*, 3576–3585.

(6) Perez, M.; Elangovan, S.; Spannenberg, A.; Junge, K.; Beller, M. Molecularly Defined Manganese Pincer Complexes for Selective Transfer Hydrogenation of Ketones. *ChemSusChem* **2017,** *10*, 83–86.

(7) Léval, A.; Agapova, A.; Steinlechner, C.; Alberico, E.; Junge, H.; Beller, M. Hydrogen production from formic acid catalyzed by a phosphine free manganese complex: investigation and mechanistic insights. *Green Chem.* **2020,** *22*, 913–920.

(8) Alberico, E.; Sponholz, P.; Cordes, C.; Nielsen, M.; Drexler, H.-J.; Baumann, W.; Junge, H.; Beller, M. Selective Hydrogen Production from Methanol with a Defined Iron Pincer Catalyst under Mild Conditions. *Angew. Chem. Int. Ed.* **2013,** *52*, 14162-14166.

(9) Werkmeister, S.; Junge, K.; Wendt, B.; Alberico, E.; Jiao, H.; Baumann, W.; Junge, H.; Gallou, F.; Beller, M. Hydrogenation of Esters to Alcohols with a Well-Defined Iron Complex. *Angew. Chem. Int. Ed.* **2014,** *53*, 8722-8726.

(10) Zhou, W.; Wei, Z.; Spannenberg, A.; Jiao, H.; Junge, K.; Junge, H.; Beller, M. Cobalt-Catalyzed Aqueous Dehydrogenation of Formic Acid. *Chem. Eur. J.* **2019,** *25*, 8459–8464.

(11) Wei, D.; Junge, H.; Beller, M. An amino acid based system for CO_2 capture and catalytic utilization to produce formates. *Chem. Sci.* **2021,** *12*, 6020–6024.

(12) Dubey, A.; Nencini, L.; Fayzullin, R. R.; Nervi, C.; Khusnutdinova, J. R. Bio-Inspired Mn(I) Complexes for the Hydrogenation of CO_2 to Formate and Formamide. *ACS Catal.* **2017,** *7*, 3864–3868.
